# OAS Deep Q-Learning-Based Fast and Smooth Control Method for Traffic Signal Transition in Urban Arterial Tidal Lanes

**DOI:** 10.3390/s24061845

**Published:** 2024-03-13

**Authors:** Luxi Dong, Xiaolan Xie, Jiali Lu, Liangyuan Feng, Lieping Zhang

**Affiliations:** 1 College of Earth Sciences, Guilin University of Technology, Guangxi Zhuang Autonomous Region, Guilin 541004, China; 2 College of Computer Science and Engineering, Guilin University of Technology, Guangxi Zhuang Autonomous Region, Guilin 541004, China; 3 Engineering and Guangxi Key Laboratory of Embedded Technology and Intelligent System, Guilin 541004, China; 4 Beijing Key Lab of Urban Intelligent Traffic Control Technology, North China University of Technology, Beijing 100144, China; 1981005@glut.edu.cn; 5 Guilin University of Technology at Nanning, Guilin University of Technology, Guilin 532100, China; 2002098@glut.edu.cn; 6 College of Mechanical and Control Engineering, Guilin University of Technology, Guangxi Zhuang Autonomous Region, Guilin 541004, China; 1994023@glut.edu.cn; 7 Key Laboratory of Advanced Manufacturing and Automation Technology, Guilin University of Technology, Guangxi Zhuang Autonomous Region, Guilin 541004, China

**Keywords:** traffic signal transition control, opening state of the tidal lane, fast and smoothing transition method, Deep Q-Learning, a reinforcement learning algorithm

## Abstract

To address traffic flow fluctuations caused by changes in traffic signal control schemes on tidal lanes and maintain smooth traffic operations, this paper proposes a method for controlling traffic signal transitions on tidal lanes. Firstly, the proposed method includes designing an intersection overlap phase scheme based on the traffic flow conflict matrix in the tidal lane scenario and a fast and smooth transition method for key intersections based on the flow ratio. The aim of the control is to equalize average queue lengths and minimize average vehicle delays for different flow directions at the intersection. This study also analyses various tidal lane scenarios based on the different opening states of the tidal lanes at related intersections. The transitions of phase offsets are emphasized after a comprehensive analysis of transition time and smoothing characteristics. In addition, this paper proposes a coordinated method for tidal lanes to optimize the phase offset at arterial intersections for smooth and rapid transitions. The method uses Deep Q-Learning, a reinforcement learning algorithm for optimal action selection (OSA), to develop an adaptive traffic signal transition control and enhance its efficiency. Finally, a simulation experiment using a traffic control interface is presented to validate the proposed approach. This study shows that this method leads to smoother and faster traffic signal transitions across different tidal lane scenarios compared to the conventional method. Implementing this solution can benefit intersection groups by reducing traffic delays, improving traffic efficiency, and decreasing air pollution caused by congestion.

## 1. Introduction

Signal transition control is a crucial aspect of traffic signal control research. A well-designed scheme can prevent significant traffic flow fluctuations, reduce delays and accidents, improve urban road traffic efficiency, and satisfy growing traffic demands. The tidal switching lane also presents a transition problem for traffic signal schemes at intersections. The signal timing transition of tidal lanes has a unique feature compared to ordinary signal timing transitions. The sequence of the signal’s phase may change before and after the tidal lane switch. The timing of the tidal lane switch in relation to the opening time of the signal transition affects the signal transition scheme. Therefore, a targeted study of the signal timing transition of tidal lanes is necessary. Such research can optimize the application of tidal lanes and increase the efficiency of traffic flow.

The optimal utilization of spatiotemporal resources in the tidal lane and its adjacent intersections is a crucial area of advancement in tidal lane research. Tidal lanes significantly affect the competence and effectiveness of adjacent intersections during switching, necessitating modifications to the intersection’s original signal timing schema. In order to avoid disruption to the normal operation of the road network when switching signal timing schemes, it is necessary to implement traffic signal transition control at intersections. The traffic signal transition control scheme facilitates the transition from one signal timing scheme to another at intersections. During the transition process, the transition scheme incorporates certain parameters from the previous timing scheme. This involves adjusting the signal phase or green light duration over one or several signal cycles to facilitate the switch to the new timing scheme. Consequently, the effectiveness of traffic control is directly affected by the traffic signal transition control scheme. Hamilton (2007) proposed an arterial fast transition method to analyze the possible impact on the original signal scheme due to signal scheme switching [1]. Pohlmann and Friedrich (2010) presented an additional transition method based on investigating transition effects in a meshed network with different coordinated relations [2]. Lee et al. (2012) proposed a non-linear mathematical model that provides constrained delay minimization through incremental and simultaneous adjustments in offset and cycle length during plan transitions [3]. Qin and Khan (2012) proposed two control strategies of traffic signal timing transition to reduce the response time and minimize the impact of emergency vehicle operation on general traffic [4]. Rita et al. (2017) presented an alternative and new mathematical model to enhance the performance of traffic signals coordination at intersections during the transition phase [5]. Zheng et al. (2017) developed an improved empirical transition method with the goal of reducing the time spent on offset correction and the offset deviations of the coordinated phases during the transition period [6]. Yao et al. (2018) established a multi-objective signal recovery transition optimization model based on the power function method [7]. Joyo et al. (2020) proposed an intelligent traffic-lights management system by exploiting relationship between traffic lights’ duration and transition pattern [8]. Zheng et al. (2021) proposed a reasonable transition method for yellow light signals and help formulate customized training programs for different types of drivers [9]. Mussa and Selekwa (2021) presented a signal timing transition period based on the quadratic optimization method [10]. Liu and Gayah (2022) proposed a travel-delay based Max Pressure model to generate lower delay with traffic transition [11]. Bouyahia et al. (2022) presented a novel prediction algorithm according to the impact of incorporation of traffic switching processes [12]. Li et al. (2022) proposed a novel approach using the FCA data for traffic congestion detection and signal transition scheme [13]. Lu et al. (2022) proposed an algebraic method of regional green wave coordinated control that can operate efficiently [14]. Storani et al. (2022) proposed a hybrid traffic flow model using signal transition to connect signalized arterial [15]. Guerra et al. (2022) compared this with a pre-timed coordinated signal control scheme, they proposed an algorithm that enabled a smooth transition to the traffic system target scheme [16]. Moradi et al. (2023) used information of connected vehicles to estimate the saturation flow rate during the transition period [17].

To effectively alleviate traffic congestion, it is vital to establish tidal lanes in a rational manner and create suitable signal control schemes. Nassiri et al. (2011) focused on the dynamic adjustment mechanism of variable lanes. A Logit choice model is used to simulate the adjustment of offline solutions, and then is extended to real-time adjustment [18]. Zhao et al. (2015) proposed a Binary-Mixed-Integer-Linear-Program (BMILP) to optimize dynamic usage of the reversible lane and signal timings [19]. Wang et al. (2016) connected traffic controllers to onboard controllers via vehicle-to-infrastructure communication to build the tidal lane signal control system [20]. Peñabaena-Niebles et al. (2020) presented an alternative and new mathematical model to enhance the performance of traffic signals coordination at intersections during the transition phase [21]. Ampountolas et al. (2019) proposed a simple and practical real-time strategy for efficient motorway tidal flow lane control [22]. Wang and Deng (2018) formulated a Mixed Network Design Problem (MDNP) to maximize the network capacity on signalized road network with reversible lanes [23]. Kotagi and Asaithambi (2019) evaluated reversible lane (tidal flow) operation using a microscopic simulation model [24]. Conceição et al. (2020) studied the problem of designing tidal lanes in a smart city with automated traffic, where both traffic assignment and tidal lanes are represented by a mixed integer nonlinear programming [25]. Di and Yang (2020) investigated demand-originated reversible lane design plans for transportation networks, where the tidal and asymmetrical characteristics of the demand structure are taken into consideration [26]. Chu et al. (2020) proposed a dynamic lane reversal-traffic scheduling management (DLR-TSM) scheme for connected and autonomous vehicles (CAVs) [27]. Wang et al. (2020) analyzed the impact of the effects before and after implementing a reversible lane [28]. Hu et al. (2021) studied the positive influence of tidal lane on relieving upstream congestion induced by a moving bottleneck and the negative effect of a moving bottleneck on a tidal lane [29]. Fu et al. (2021) developed a direction-changeable lane-based cell transmission model to find an optimal DLR scheme for a roadway segment with stochastic traffic flow in both directions [30]. Liu et al. (2022) presented a predictive empowered assignment scheme for reversible lane (PEARL), which integrates the advanced traffic flow prediction module and bi-level optimization model [31]. Peng and Wang (2022) proposed a coordinated control model for arterials with asymmetric traffic demands in both oversaturated and unsaturated directions [32]. Zhou et al. (2023) proposed a real-time dynamic reversible lane safety control model based on the length of reversible lanes and the transition of signal timings [33]. Franzl et al. proposed a traffic-light-like system that enables the local grid operator to trigger situation-aware customer behavior, supporting grid stability when needed and, in return, allowing customers to fully exploit temporary grid capacity when no safety or stability issues persist [34]. Ma et al. presented a method on the development and evaluation of an economic-driving assistance program for transit vehicles (EDTVs) which can minimize energy consumption [35]. Park et al. presented the development of a sustainable traffic signal control system and speed management framework consisting of a microscopic simulation model, a microscopic fuel consumption and emission model, and a genetic algorithm–based optimizer [36]. Huang et al. developed an adaptive traffic signal control method with equilibrium constraints under stochastic demand. The methodology was developed to model transportation network design with signal settings in the presence of demand uncertainty [37]. Wang et al. developed a stochastic dynamic model for urban traffic flow subject to practical constraint characteristics of intersections equipped with traffic light [38].

In summary, most studies have focused on addressing signal transition issues at intersections and have suggested various solutions. The studies differ in range for adjusting relevant parameters for transition signals. The studies on transitioning signal schemes mainly focus on adjusting the transition step and adapting the switching sequence through phase offset. However, research on strategies for controlling signal transitions in different traffic scenarios is lacking. Each scenario requires distinct control methods. For instance, in a tidal lane scenario, opening the lane triggers changes in the phase sequence of both old and new phase schemes. Additionally, the effectiveness of intersection signal control is affected by the opening time of the tidal lane and the signal transition control. The conventional method for adjusting transitions neglects the circumstance of the transition start being located solely at the conclusion of the previous cycle. This results in the failure of the conventional signal transition control method to consider the actual traffic states of the intersection, which limits its application. In the tidal lane scenario, the transition periods of different intersections may diverge. The conventional method only considers the transition within the same time period, resulting in a less than optimal control effect of the signal transition scheme. To achieve globally optimal traffic control, it is important to consider these factors. Furthermore, most studies on traffic control in tidal lanes do not take into account the potential alteration of signal control methods due to tidal lane switching before and after. Furthermore, existing studies often focus on only one section of the tidal lane, with limited research on how to regulate signals when opening the tidal lane at different times for multiple connected upstream and downstream sections. Therefore, it is essential to enhance the actual impact of its implementation.

To maintain objectivity, this study has chosen to focus on traffic flow due to its stability during tidal lane function switching. The traffic flow is segmented based on different flow directions. The relationship between signal transition time and tidal lane opening time is then analyzed using an objective function, constraints, and a signal transition control model through the phase-sequence design methodology. In the scenario of tidal lanes, it is crucial to note that the tidal lanes at each intersection may have varying opening times. This necessitates an examination of synchronous and asynchronous signal transitions at adjacent intersections. A model for controlling signal transition in arterial coordinated networks has been developed to achieve smooth phase offset transitions. The Deep Q-Learning algorithm is used for optimal action selection (OSA) in order to obtain a global optimal solution. This optimizes traffic signal transitions for tidal lanes.

This paper is organized as follows. The overall framework is shown in Section 2. The design of the intersection overlap phase scheme based on the traffic flow conflict matrix is proposed in Section 3. Section 4 presents the analysis of signal transition at key intersections based on the flow ratio. The model of the arterial coordinated signal transition is established in Section 5. Section 6 carries out the solution algorithm. The results of the case studies are shown in Section 7. Section 8 presents the conclusions.

## 2. Overall Framework

**Figure 1:**
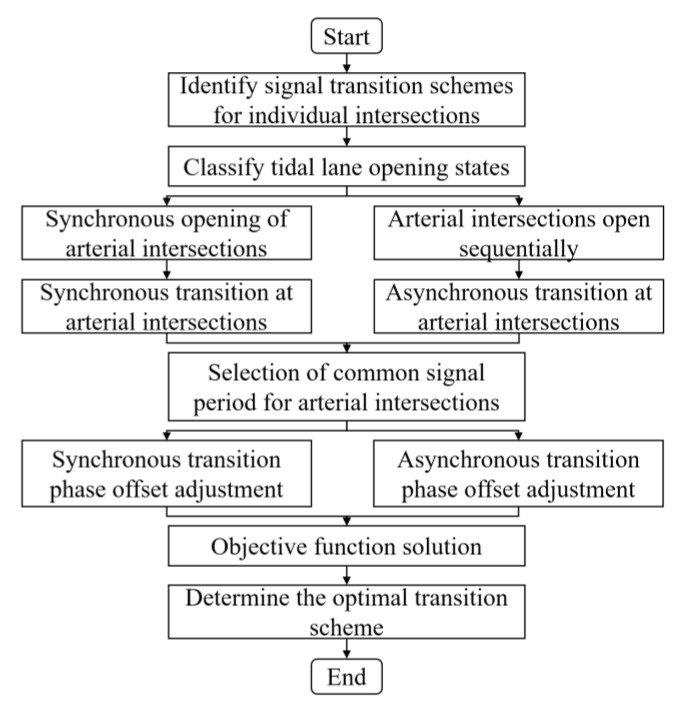
The flowchart of arterial intersection transition.

The methodology for controlling traffic signal transitions on tide lane-oriented arterial coordination differs from research on ordinary arterial coordination. This is due to the varying times at which different intersections with tidal lanes are open. Therefore, discussion and analysis are necessary to determine how to handle transitions that cannot be completed within the same time period at arterial intersections. These cases can be classified as either synchronous or asynchronous signal transitions, as illustrated in Figure 1.

The objective of this paper is to identify the most appropriate signal transition control model for arterial coordination, while satisfying the constraints, to ensure traffic flow stability during arterial traffic signal transition in a tidal lane scenario. This paper examines the optimal signal transition control model for adjacent intersection tidal lanes with varying opening states. Next, the signal transition period and phase offset are optimized using phase offset smoothing transition as the optimal control objective. Then, a function is established to determine the final arterial coordinated signal transition scheme. Due to imbalanced traffic flow in both directions at the arterial intersection in the tidal lane scenario, a coordinated one-way control method is recommended.

## 3. The Design of the Intersection Overlap Phase Scheme Based on the Traffic Flow Conflict Matrix

This paper focuses on arterial signal intersections with independent right-turning traffic flow. At these intersections, the right-turning traffic does not have separate signal control. However, the effect of the right-turning traffic flow on the original straight direction of traffic flow on the road it occupies and the straight traffic flow in the lane it turns into does not need to be taken into account. Under the given setting conditions, the flow direction grouping can be divided into eight sections, which can be represented by the vector *U* in Equation (1).

(1) $$U={\left(NT,NL,ET,EL,ST,SL,WT,WL\right)}^{T}$$

where *NT* represents straight traffic flow in the north direction at the intersection. *NL* represents left traffic flow in the north direction at the intersection. *ET* represents straight traffic flow in the east direction at the intersection. *EL* represents left traffic flow in the east direction at the intersection. *ST* represents straight traffic flow in the south direction at the intersection. *SL* represents left traffic flow in the south direction at the intersection. *WT* represents straight traffic flow in the west direction at the intersection. *WL* represents left traffic flow in the west direction at the intersection.

From the perspective of traffic flow, a traffic conflict matrix is defined by Equations (2) and (3) based on the composition of traffic flow.

(2) $$M=s_{m,n}=UU^{T}$$

(3) $$s_{m,n}=\left\{\begin{array}{ll} 1 & conflict\\ 0 & non\;conflict \end{array}\right.$$

where *s_m,n_* represents two states of traffic flow, the values are taken in the range of 0 or 1, where 1 is a conflicting state, and 0 is a non-conflicting state.

The phase sequence of the signal transition scheme in the tidal lane scenario is determined by the traffic conflict matrix. The tidal lane is designed for straight traffic flow, so if the straight traffic flow in each direction is in the same phase as the left-turning traffic flow, it will result in a conflict. Hence, it cannot be set in the same phase. The traffic conflict matrix is calculated using Equation (4).

(4) $$M=\left[\begin{array}{llllllll} 0 & 1 & 1 & 0 & 0 & 1 & 1 & 1\\ 1 & 0 & 1 & 1 & 1 & 0 & 0 & 1\\ 1 & 1 & 0 & 1 & 1 & 0 & 0 & 1\\ 0 & 1 & 1 & 0 & 1 & 1 & 1 & 0\\ 0 & 1 & 1 & 1 & 0 & 1 & 1 & 0\\ 1 & 0 & 0 & 1 & 1 & 0 & 1 & 1\\ 1 & 0 & 0 & 1 & 1 & 1 & 0 & 1\\ 1 & 1 & 1 & 0 & 0 & 1 & 1 & 0 \end{array}\right]$$

The tidal lanes are divided into different directions based on the traffic conflict matrix. The sequence of the overlap phase for both symmetrical release and separate release is designed in Figure 2, Figure 3, Figure 4, Figure 5, Figure 6, Figure 7, Figure 8 and Figure 9.

(1) Tidal lanes for NT traffic flow

**Figure 2:**
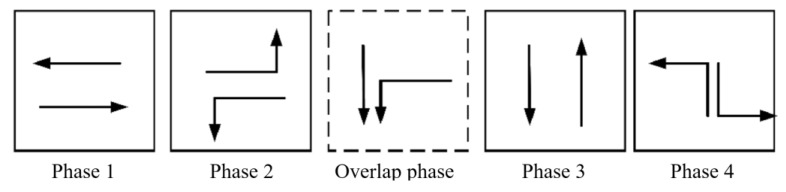
Overlap phase of NT traffic flow under symmetrical release.

**Figure 3:**
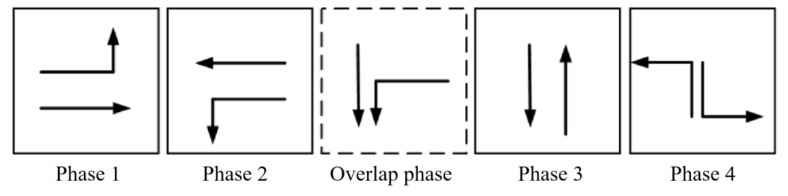
Overlap phase of NT traffic flow under separate release.

(2) Tidal lanes for ST traffic flow

**Figure 4:**
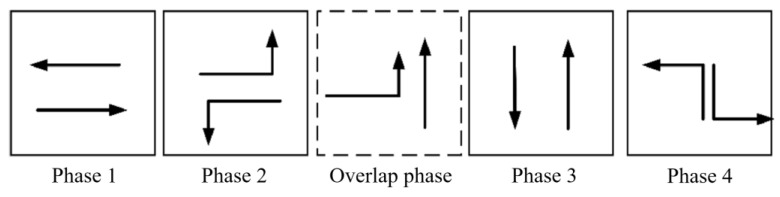
Overlap phase of ST traffic flow under symmetrical release.

**Figure 5:**
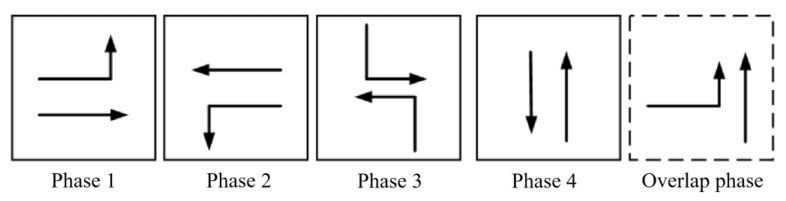
Overlap phase of ST traffic flow under separate release.

(3) Tidal lanes for WT traffic flow

**Figure 6:**
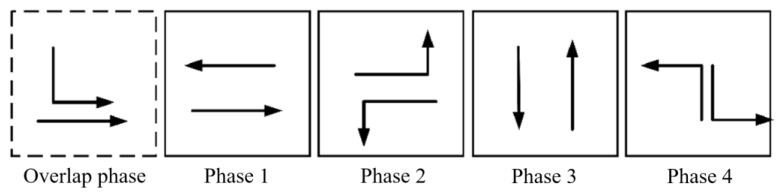
Overlap phase of WT traffic flow under symmetrical release.

**Figure 7:**
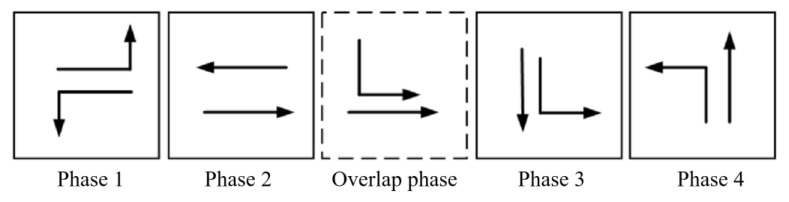
Overlap phase of WT traffic flow under separate release.

(4) Tidal lanes for ET traffic flow

**Figure 8:**
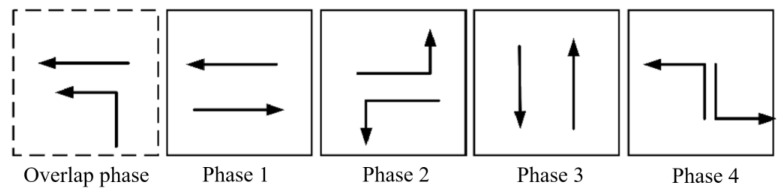
Overlap phase of ET traffic flow under symmetrical release.

**Figure 9:**
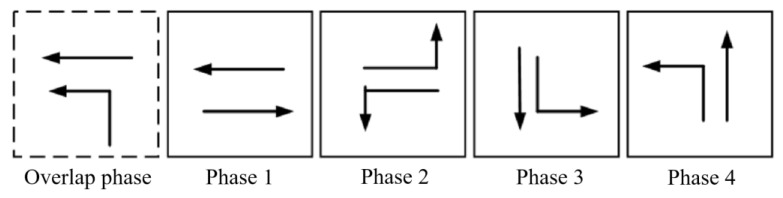
Overlap phase of ET traffic flow under separate release.

The aforementioned content outlines the organization of tidal lanes to ensure smooth traffic flow in different directions, using an overlap phase scheme. The decision between a symmetrical or separate release is determined by the initial signal scheme.

## 4. The Analysis of Signal Transition at Key Intersection Based on the Flow Ratio

**Figure 10:**
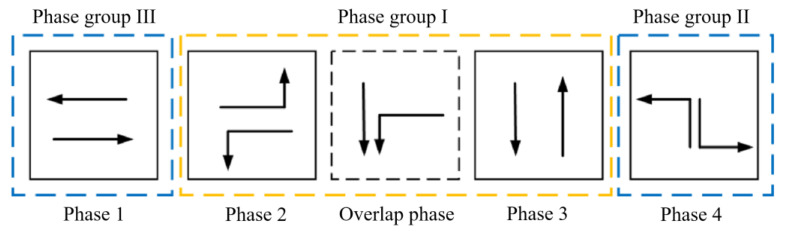
Schematic diagram of overlap phase group.

The design of the post-transition signal scheme is the first step, taking into account the change in the phase scheme of the phase sequence. Next, the signal transition scheme is determined. The phases before and after the overlap phase are considered phase group I, while the remaining phases are designated as phase group II and phase group III. In the case of tidal lanes with symmetric release scheme for *NT* traffic flow, the key traffic flow combination in phase group I consists of *NT* + *WL* or *ST* + *EL*. The key traffic flow combination in phase group II comprises *NL* or *SL*, and in phase group III, it is *WT* or *ET*, as stated by Peng and Zhang (2001) [39]. The schematic diagram of overlap phase group is shown in Figure 10.

The flow ratio of the phases in phase group I (defined as $y_{I}^{\prime }$) is calculated using Equation (5).

(5) $$y_{I}^{\prime }=\max(y_{NT}+y_{WL},y_{ST}+y_{EL})$$

where $y_{NT}$ is the flow ratio of *NT*, $y_{WL}$ is the flow ratio of *WL*. $y_{ST}$ is the flow ratio of *ST*, $y_{EL}$ is the flow ratio of *EL*. As stated by Peng and Zhang (2001) [39], the key traffic flow combination should be the one with the higher flow ratio in $y_{NT}+y_{WL}$ or $y_{ST}+y_{EL}$.

The flow ratio of the phases in phase group II (defined as $y_{II}^{\prime }$) is calculated using Equation (6).

(6) $$y_{II}^{\prime }=\max(y_{NL},y_{SL})$$

where $y_{NL}$ is the flow ratio of *NL*, $y_{SL}$ is the flow ratio of *SL*. As stated by Peng and Zhang (2001) [39], the key traffic flow combination should be the one with the higher flow ratio in $y_{NL}$ or $y_{SL}$.

The flow ratio of the phases in phase group III (defined as $y_{III}^{\prime }$) is calculated using Equation (7).

(7) $$y_{III}^{\prime }=\max(y_{WT},y_{ET})$$

where $y_{WT}$ is the flow ratio of *WT*. $y_{ET}$ is the flow ratio of *ET*. As stated by Peng and Zhang (2001) [39], the key traffic flow combination should be the one with the higher flow ratio in $y_{WT}$ or $y_{ET}$.

Set the signal cycle as *C_k_*. The total loss time is *L*. The effective green time is *G_e_*__*k*_. The total flow ratio is *Y_k_*. *k* represents the *k*th transition period.

(8) $$C_{k}=\frac{1.5L+5}{1-Y_{k}}$$

(9) $$Y_{k}=\sum_{i=1}^{3}y_{i}=y_{I}^{\prime }+y_{II}^{\prime }+y_{III}^{\prime }$$

(10) $$G_{e\_k}=C_{k}-L$$

Equations (11) to (13) calculate the effective green time for phase groups I, II, and III.

(11) $$g_{I\_k}=\frac{y_{I\_k}^{\prime }}{Y_{k}}G_{e\_k}$$

(12) $$g_{II\_k}=\frac{y_{II\_k}^{\prime }}{Y_{k}}G_{e\_k}$$

(13) $$g_{III\_k}=\frac{y_{III\_k}^{\prime }}{Y_{k}}G_{e\_k}$$

where *k* represents the *k*th transition period. $g_{I\_k}$ is the effective green time for phase group I at the *k*th transition period. $g_{II\_k}$ is the effective green time for phase group II at the *k*th transition period. $g_{III\_k}$ is the effective green time for phase group III at the *k*th transition period. $y_{I\_k}^{\prime }$ is the flow ratio of phase group I at the *k*th transition period. $y_{II\_k}^{\prime }$ is the flow ratio of phase group II at the *k*th transition period. $y_{III\_k}^{\prime }$ is the flow ratio of phase groups III at the *k*th transition period.

As the initial group of phases consists of three phases, signal timing is required for each individual phase.

(1) If $y_{NT\_k}+y_{WL\_k}>y_{ST\_k}+y_{EL\_k}$, it satisfies the following conditions.

(14) $$g_{I_{1}\_k}=\frac{y_{WL\_k}}{y_{NT\_k}+y_{WL\_k}}g_{I\_k}$$

(15) $$g_{I_{2}\_k}=\frac{y_{NT\_k}-y_{ST\_k}}{y_{NT\_k}+y_{WL\_k}}g_{I\_k}$$

(16) $$g_{I_{3}\_k}=\frac{y_{ST\_k}}{y_{NT\_k}+y_{WL\_k}}g_{I\_k}$$

(2) If $y_{ST\_k}+y_{EL\_k}>y_{NT\_k}+y_{WL\_k}$, it satisfies the following conditions.

(17) $$g_{I_{1}\_k}=\frac{y_{WL\_k}}{y_{ST\_k}+y_{EL\_k}}g_{I\_k}$$

(18) $$g_{I_{2}\_k}=\frac{y_{EL\_k}-y_{WL\_k}}{y_{ST\_k}+y_{EL\_k}}g_{I\_k}$$

(19) $$g_{I_{3}\_k}=\frac{y_{ST\_k}}{y_{ST\_k}+y_{EL\_k}}g_{I\_k}$$

Accordingly, traffic flow plays an essential role in determining signal timing. The traffic flow ratio increases progressively during peak morning and evening periods, serving as a direct reflection of these changes. Therefore, this paper proposes the change coefficient of the traffic flow ratio (defined as *ε*). To design the signal transition control scheme, *ε_m_* is used to represent the change in the ratio of traffic flow for the m-traffic prior to and post-transition, whereas Δ*ε_m_* represents the change in the ratio of traffic flow for the *m*-traffic before and after two transition cycles.

(20) $$\varepsilon_{m}=\frac{y_{m\_B}}{y_{m\_A}}$$

(21) $$\Delta \varepsilon_{m}=\frac{\varepsilon_{m}-1}{N+1}$$

(22) $$y_{m\_k}=y_{m\_(k-1)}\times [1+\left(\Delta \varepsilon_{m}\pm 0.01\right)]$$

where $y_{m\_B}$ represents the traffic flow ratio of the post-transition stream *m*, $y_{m\_A}$ represents the traffic flow ratio of the pre-transition stream *m*, *N* represents the number of cycles of signal transition, *N* + 1 indicates the number of changes from the pre-transition to the post-transition cycle. Additionally, $y_{m\_k}$ represents the traffic flow ratio of the m-stream of the *k*th transition cycle. $y_{m\_(k-1)}$ represents the traffic flow ratio of the m-stream of the *k* − 1th transition cycle. $y_{m\_A}$ equals $y_{m\_0}$ when *k* = 1 (usually, the traffic flow ratio is taken with an accuracy of 0.01, therefore, the $\Delta \varepsilon_{m}$ adjustable range is adopted as 0.01).

Thus, the signal transition period is calculated using Equations (23) and (24) as follows.

(23) $$C_{k}^{\prime }=\frac{1.5L+5}{1-Y_{k}^{\prime }}$$

(24) $$Y_{k}=\sum_{i=1}^{3}y_{ki}$$

where $C_{k}^{\prime }$ represents the signal cycle in the *k*th transition cycle. $Y_{k}^{\prime }$ represents the total traffic ratio in the *k*th transition cycle. $y_{ki}$ represents the traffic ratio of the *i*th phase group in the *k*th transition cycle, where $k\in \left(1,N\right)$.

The total number of transition cycles *N* is determined by the total transition duration, *O*(*s*), which can be adjusted based on the actual situation. The duration is calculated using Equation (25).

(25) $$\frac{O}{C_{A}}\leq N\leq \frac{O}{C_{B}}$$

where $C_{A}$ represents the pre-transition signal cycle. $C_{B}$ represents the post-transition signal cycle.

### 4.1. The Key Intersection for Calculating the Queue Length

The queue length for vehicles at the intersection (defined as *L_j_*) is the total of the average number of stationary vehicles and the number of vehicles that are held up during the current red light, as well as the number of vehicles in the queue resulting from the approaching cycle [40,41,42].

(26) $$L_{j}=L_{Rj}+L_{0j}$$

(27) $$L_{Rj}=\left\{\begin{array}{ll} q_{k}R_{j} & \left(x<1\right)\\ QR_{j} & \left(x\geq 1\right) \end{array}\right.$$

(28) $$L_{0j}=\left\{\begin{array}{ll} \frac{QC_{k}}{4}\left[x-1+\sqrt{{\left(x-1\right)}^{2}+\frac{4x}{QC_{k}}}\right] & \left(x>X_{0}\right)\\ 0 & \left(x\leq X_{0}\right) \end{array}\right.$$

(29) $$L={\sum_{j=1}^{m}\left(L_{j}-\frac{\sum_{j=1}^{m}L_{j}}{m}\right)}^{2}$$

where *L_j_* is the vehicle queue length during *j*th cycle. *L_Rj_* is the number of backlogged vehicles during the red-light period. *L*_0*j*_ is the average number of stopped vehicles in all cycles. *L* is the variance of the queue length for each stream of vehicles. *q_k_* is the vehicle reach flow rate in cycle *k*. *R_j_* is red-light time. *Q* is capacity. *C_k_* is the transition signal cycle for the *k*th transition phase in the transition scheme. *x* is the saturation. *X*_0_ is the saturation threshold.

### 4.2. The Key Intersection for Calculating the Average Delay

The research applies the conventional Webster model to determine the average vehicle delay [43,44]. The vehicle delay model is calculated using Equation (30).

(30) $$D_{j}=0.9\left[\frac{C{\left(1-\lambda_{j}\right)}^{2}}{2(1-\lambda_{j}x_{j})}+\frac{x_{j}^{2}}{2q_{j}(1-x_{j})}\right]$$

Equation (30) depicts the average delay experienced by a vehicle in a cycle.

(31) $$\bar{D}=\frac{\sum_{j=1}^{m}D_{j}q_{j}}{\sum_{j=1}^{m}q_{j}}$$

where *D_j_* represents the delay of the *j*th flow as it passes through the intersection during a single cycle. $\bar{D}$ is the average vehicle delay. $\lambda_{j}$ indicates the green time ratio for the *j*th flow. $x_{j}$ is the saturation of the vehicle flow. $q_{j}$ represents traffic volume for the *j*th stream. *C* represents the signal cycle.

### 4.3. Signal Transition Objective Function for the Key Intersection

This paper focuses on developing a function for designing a single intersection signal transition control method for tidal lanes, with the aim of improving traffic flow. The queue length and average delay performance indices are adopted as optimal control objectives. The aim is to promote smooth traffic at the intersection.

This paper presents the concept of queue length equalization control. The aim is to reduce the difference between queue lengths of various streams at the intersection by allocating more green time to streams with longer queues. This approach ensures smooth and rapid signal transitions, making it more suitable for tidal lane scenarios. The objective function is outlined in Equation (32).

(32) $$Y=\min\left\{\mu L+\eta \bar{D}\right\}$$

where *Y* is the objective function. *L* is the variance of the queue length for each stream of vehicles. $\bar{D}$ is the average vehicle delay.

The constraints on the objective function are displayed in Equation (33) as follows.

(33) $$s.t.\left\{\begin{array}{l} C_{\min}\leq C_{k}\leq C_{\max}\\ C_{k}=C_{k-1}+\Delta C_{k}\\ C_{A}-C_{B}=\sum_{k=1}^{n}\Delta C_{k}\\ \sum_{i=1}^{n}g_{i}\leq C_{k}-\gamma \\ g_{i\min}\leq g_{i}\leq g_{i\max}\\ g_{ik}=g_{i(k-1)}+\Delta g_{ik}\\ y_{jk}=\frac{q_{jk}}{\left(n_{j}+\alpha \right)Sb}\\ x\leq 0.9\\ \mu +\eta =1 \end{array}\right.$$

where *g_i_* is the green light time at *i*th phase. γ is the total loss time at the intersection. *y_jk_* is traffic flow ratio during the transition cycle *k* for stream *j*. *n_j_* is the number of lanes for stream *j*. *q_jk_* is traffic volume during the transition cycle *k* for stream *j*. *S_b_* is saturated flow. μ and η are non-negative weighting factors.

## 5. The Model of Arterial Coordinated Signal Transition

### 5.1. Analysis of Tidal Lanes at Arterial Intersections

The opening state of tidal lanes at arterial intersections can impact the intersection signaling scheme and traffic flow ratio, subsequently affecting the signal transition scheme. Therefore, it is necessary to analyze the opening state of each intersection in the arterial line. Tidal lanes at intersections of arterials may open synchronously or asynchronously. The opening situation of the arterial intersection tidal lane is classified into two categories: synchronous opening and asynchronous opening. The value α = l represents the opening of the tidal lane, while the value α = 0 indicates its closure. Table 1 and Table 2 provide an example of four intersections and three transition cycles.

### 5.2. Analysis of Tidal Lanes at Arterial Intersections

Optimizing the phase offset at each intersection can render the arterial coordinated signal transition scheme for tidal lanes more appropriate for signal control at tidal lane intersections. As the opening states of tidal lanes at adjacent intersections can impact traffic flow and convergence may occur, it is necessary to recalculate the phase offset optimally. This will ensure that the signal transition scheme is better suited for controlling tidal lane scenarios.

Phase offset includes relative offset and absolute offset [5,45,46]. In practical coordination of traffic control, relative offset is commonly utilized. This refers to the time that passes between the start of a green light or the end of a red light at two adjacent intersections in an arterial intersection. The symbol (*ζ*) represents this offset, which can be measured either in seconds (s) or as a percentage of the cycle length. The phase offset calculation equation in Webster‘s model is defined as Equations (34) and (35).

(34) $$\zeta =\frac{s}{v}\times 3600$$

(35) $$\varphi_{\left(h,h-1\right)}=\mathrm{mod}(\frac{T_{tra}}{C})$$

where $\varphi_{\left(h,h-1\right)}$ represents the relative offset between adjacent intersections. $T_{tra}$ is the duration of vehicle travel. *v* represents the average speed of the vehicle. s represents the distance between adjacent intersections. *C* is the signal cycle.

**Figure 11:**
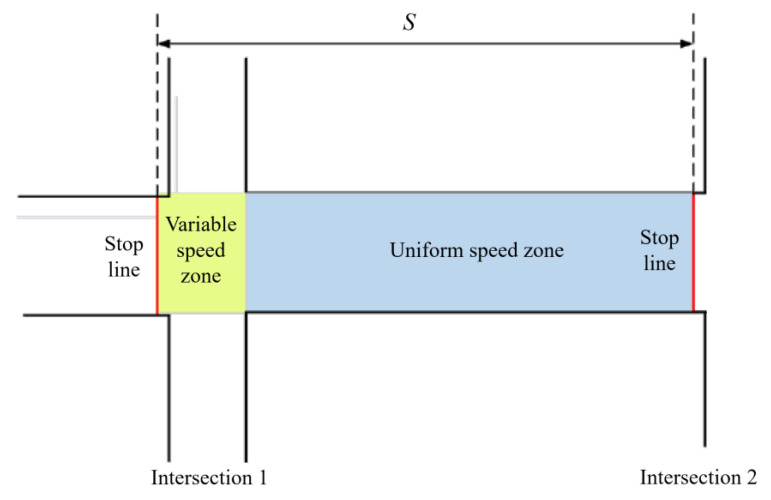
Schematic diagram of vehicle speed at intersection.

The phase offset has been adjusted to improve signal control effectiveness based on the traffic flow characteristics of the road section, as illustrated in Figure 11.

(1) The tidal line at both intersection 1 and intersection 2 is open.

Scenario 1: When vehicles reach the intersection stop line at a red light, they must come to a complete stop. After stopping, vehicles should accelerate to the designated speed before continuing at a constant speed.

(36) $$\left\{\begin{array}{l} v_{2}=at_{cha}\\ s_{cha}=\frac{1}{2}at_{cha}^{2} \end{array}\right.$$

(37) $$t_{cha}=\frac{2s_{cha}}{v_{2}}$$

where *v*_2_ represents the design speed of the road section. *t_cha_* refers to the travel time in the variable speed zone. *s_cha_* indicates the distance of the speed zone, which represents the length of the intersection. *a* represents the acceleration of the vehicle in the variable speed zone.

(38) $$t_{uni}=\frac{s_{uni}}{v_{2}}$$

(39) $$s=s_{uni}+s_{cha}$$

(40) $$T_{tra}=t_{uni}+t_{cha}=\frac{s_{uni}+s_{cha}}{v_{2}}$$

where *t_uni_* represents the travel time in the uniform speed zone, and *s_uni_* represents the distance of the uniform speed zone.

Scenario 2: When vehicles arrive at the intersection stop line and encounter a green light, it is possible to pass through the intersection at a uniform speed.

(41) $$T_{tra}=\frac{s}{v_{2}}$$

(2) The tidal line at intersection 1 is open, while at intersection 2, the tidal line is not open.

Scenario 1: When vehicles approach an intersection and encounter a red light at the stop line, they must come to a complete stop. After stopping, vehicles should accelerate to the designated speed and proceed at a constant speed. This process is the same as at adjacent intersections where tidal lanes are accessible.

Scenario 2: When the green light appears, vehicles must come to a stop at the intersection stop line. As the number of lanes decreases, vehicles are required to slow down within the intersection’s variable speed zone before accelerating into the road section. It is assumed that in the variable speed zone, the first half of the traffic flow decelerates, and the second half accelerates.

**Figure 12:**
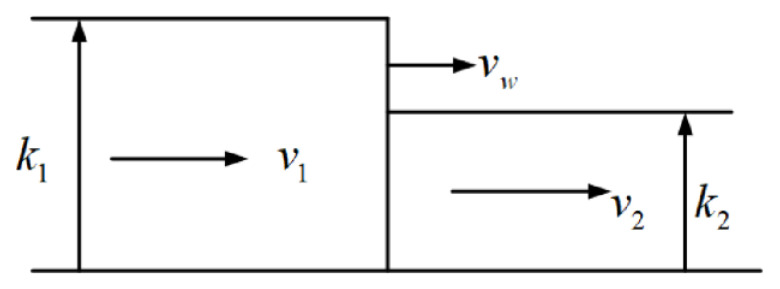
The flowchart of traffic wave analysis.

The traffic flow can be known based on the continuity equation for traffic flow and the traffic flow model [47], as illustrated in Figure 12.

(42) $$\left\{\begin{array}{l} q_{1}=k_{1}v_{1}\\ q_{2}=k_{2}v_{2} \end{array}\right.$$

where *q*_1_ and *q*_2_ represent the traffic flow of intersection 1 and 2 respectively. The traffic density of intersection 1 and 2 is represented by *k*_1_ and *k*_2_ respectively. *v*_1_ refers the design speed of the road section of the intersection.

(43) $$v_{w}=\frac{q_{1}-q_{2}}{k_{1}-k_{2}}$$

where *v_w_* is the speed of the intersection.

*t_dec_* is determined using the displacement equation ($\frac{1}{2}s_{cha}=\frac{v_{1}+v_{w}}{2}\cdot t_{dec}$).

(44) $$t_{dec}=\frac{s_{cha}}{v_{1}+v_{w}}$$

Similarly

(45) $$t_{acc}=\frac{s_{cha}}{v_{2}+v_{w}}$$

(46) $$t_{cha}=t_{acc}+t_{dec}$$

(47) $$T=t_{cha}+t_{uni}=\frac{s_{cha}}{v_{1}+v_{w}}+\frac{s_{cha}}{v_{2}+v_{w}}+\frac{s_{uni}}{v_{2}}$$

If vehicles arrive at the intersection during the same green light time, the phase offset should be satisfied $\varphi +\lambda C\leq T_{tra}$. Additionally, the time for the last vehicle to reach the downstream intersection should be satisfied $g_{i-1}+T_{tra}$. Finally, the phase offset should be satisfied $\varphi +g_{i}+\lambda C\geq g_{i-1}+T_{tra}$ when the last vehicle reaches the downstream stop line. In order to ensure smooth traffic flow through the downstream intersection, the phase offset must satisfy the condition presented in (48).

(48) $$g_{h}-g_{h+1}+T_{tra}-\lambda C\leq \varphi_{\left(h,h-1\right)}\leq T_{tra}-\lambda C$$

### 5.3. Analysis of Signal Transition at Arterial Intersections

The opening of tidal lanes at each intersection affects traffic flow and signal cycle, which differs from traditional arterial coordinated control. Therefore, this paper will analyze the transition signal cycle scheme in relation to the enabled tidal lanes. The intersection with the highest total flow ratios at each intersection, which is typically the intersection with the longest signal cycle, is considered the key intersection for coordinated control of arterial roads. The signal transition scheme for the arterial road is established using the single-intersection signal transition model. Different transition schemes for each intersection are considered to establish the key intersections for each signal cycle. Once this is done, the arterial signal transition scheme is established. Table 3 shows the calculation results of signal transition cycle.

The signal transition cycle is calculated using Equation (49).

(49) $$\left\{\begin{array}{l} C_{1}=\max\left\{C_{11},C_{21},\cdots ,C_{n1}\right\}\\ C_{2}=\max\left\{C_{12},C_{22},\cdots ,C_{n2}\right\}\\ \cdots \\ C_{k}=\max\left\{C_{1k},C_{2k},\cdots ,C_{nk}\right\} \end{array}\right.$$

where *C_nk_* is the kth transition cycle of the *n*th intersection. *C_k_* represents the *k*th transition cycle.

#### 5.3.1. Synchronous Transition

The tidal lanes are opened simultaneously at each intersection, and the signal transition scheme is implemented accordingly. For example, the three signal transition cycles at three intersections are synchronized. Table 4 presents the synchronous transition for signal scheme.

**Figure 13:**
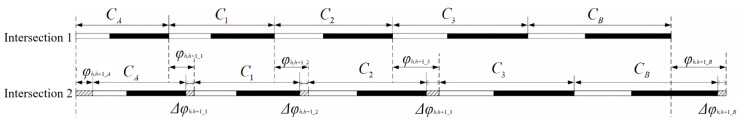
Phase offset adjustment for synchronous transitions.

The phase offset for the synchronous transition is adjusted as follows, which is shown in Figure 13.

(50) $$\left\{\begin{array}{l} \varphi_{h,h+1\_1}=\varphi_{h,h+1\_A}+\Delta \varphi_{h,h+1\_1}\\ \varphi_{h,h+1\_2}=\varphi_{h,h+1\_1}+\Delta \varphi_{h,h+1\_2}\\ \varphi_{h,h+1\_3}=\varphi_{h,h+1\_2}+\Delta \varphi_{h,h+1\_3}\\ \varphi_{h,h+1\_B}=\varphi_{h,h+1\_3}+\Delta \varphi_{h,h+1\_B} \end{array}\right.$$

(51) $$\begin{array}{l} \Delta \varphi =\varphi_{h,h+1\_B}-\varphi_{h,h+1\_A}\\ =\Delta \varphi_{h,h+1\_1}+\Delta \varphi_{h,h+1\_2}+\Delta \varphi_{h,h+1\_3}+\Delta \varphi_{h,h+1\_B} \end{array}$$

where $\varphi_{h,h+1\_A}$ represents, the phase offset between intersection *h* + 1 and intersection *h* in the pre-transition cycle. $\varphi_{h,h+1\_1}$, $\varphi_{h,h+1\_2}$, and $\varphi_{h,h+1\_3}$ represent the phase offsets between intersection *h* + 1 and intersection *h* in transition cycles I, II and III, respectively. $\varphi_{h,h+1\_B}$ represents the phase offset between intersection *h* + 1 and intersection *h* in the post-transition cycle. $\Delta \varphi_{h,h+1\_1}$, $\Delta \varphi_{h,h+1\_2}$, $\Delta \varphi_{h,h+1\_3}$ and $\Delta \varphi_{h,h+1\_B}$ represent the adjustments of the phase offset between intersection *h* + 1 and intersection *h* in different cycles. The change in the phase offset between before and after the transition is represented by Δφ.

#### 5.3.2. Asynchronous Transition

During asynchronous transition, the tidal lanes on each road section of the arterial are opened sequentially. Additionally, the signal transition cycle is implemented sequentially at each intersection. The scheme for the asynchronous transition signal cycle is shown in Table 5.

**Figure 14:**
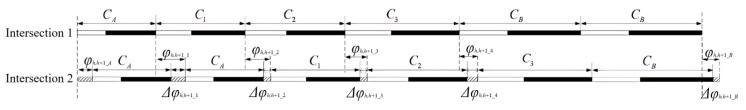
Phase offset adjustment for asynchronous transitions.

The phase offset for the asynchronous transition is adjusted as follows, which is shown in Figure 14.

(52) $$\left\{\begin{array}{l} \varphi_{h,h+1\_1}=\varphi_{h,h+1\_A}+\Delta \varphi_{h,h+1\_1}\\ \varphi_{h,h+1\_2}=\varphi_{h,h+1\_1}+\Delta \varphi_{h,h+1\_2}\\ \varphi_{h,h+1\_3}=\varphi_{h,h+1\_2}+\Delta \varphi_{h,h+1\_3}\\ \varphi_{h,h+1\_4}=\varphi_{h,h+1\_4}+\Delta \varphi_{h,h+1\_4}\\ \varphi_{h,h+1\_B}=\varphi_{h,h+1\_3}+\Delta \varphi_{h,h+1\_B} \end{array}\right.$$

(53) $$\begin{array}{ll} \Delta \varphi & =\varphi_{h,h+1\_B}-\varphi_{h,h+1\_A}\\ & =\Delta \varphi_{h,h+1\_1}+\Delta \varphi_{h,h+1\_2}+\Delta \varphi_{h,h+1\_3}+\Delta \varphi_{h,h+1\_4}+\Delta \varphi_{h,h+1\_B} \end{array}$$

### 5.4. Arterial Coordinated Signal Transition Objective Function

**Figure 15:**
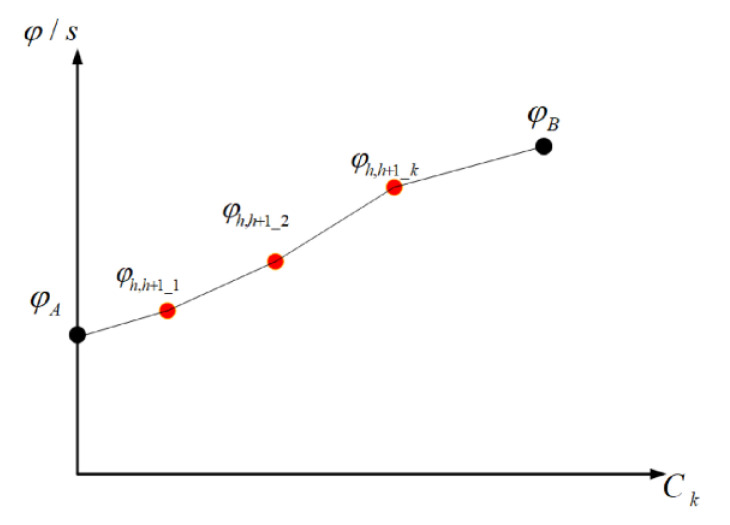
Schematic diagram of phase offset transition.

Signal transition is the process by which each intersection gradually reduces the amount of phase offset change to zero through signal transition. Directly switching the phase offset at the intersection from the flat (peak) hour to the peak (flat) hour may cause unstable traffic flow and lead to poor intersection access efficiency. To ensure a smooth and rapid transition of the phase offset at intersections, we developed a model for smooth phase offset transition by analyzing the curve of phase offset change. The objective function for arterial coordinated signal control is designed to achieve a smooth phase offset transition. The schematic diagram of phase offset transition is shown in Figure 15.

(54) $$\min\theta =\sum_{k=1}^{N+1}\sum_{h}^{n}\left|\theta_{h,h+1\_k+1}-\theta_{h,h+1\_k}\right|$$

(55) $$\left\{\begin{array}{l} \theta_{h,h+1\_1}=\left|\frac{\Delta \varphi_{h,h+1\_1}}{C_{A}}\right|\\ \theta_{h,h+1\_2}=\left|\frac{\Delta \varphi_{h,h+1\_2}}{C_{1}}\right|\\ \begin{array}{l} \cdots \\ \theta_{h,h+1\_k}=\left|\frac{\Delta \varphi_{h,h+1\_k}}{C_{k-1}}\right| \end{array}\\ \theta_{h,h+1\_B}=\left|\frac{\Delta \varphi_{h,h+1\_B}}{C_{B}}\right| \end{array}\right.$$

(56) $$s.t.\left\{\begin{array}{l} C_{\min}\leq C_{k}\leq C_{\max}\\ C_{k}=C_{k-1}+\Delta C_{k}\\ C_{A}-C_{B}=\sum_{k=1}^{n}\Delta C_{k}\\ g_{h-1}-g_{h}+T_{tra}-\lambda C_{k}\leq \varphi_{h,h+1}\leq T_{tra}-\lambda C_{k}\\ \frac{O}{C_{A}}\leq N\leq \frac{O}{C_{B}}\\ \Delta \varphi =\sum_{k=1}^{n}\Delta \varphi_{h,h+1\_k}\\ \Delta \varphi_{h,h+1\_k}=\varphi_{h,h+1\_k}-\varphi_{h,h+1\_(k-1)} \end{array}\right.$$

where *g_h_* is the green time for the coordinated phase of the *h*th intersection. $\varphi_{h,h+1\_k}$ is the phase offset between the *h*th intersection and the *h* − 1th intersection. Δφ represents the value of the phase offset between the *h*th and *h* − 1th intersection before and after the signal transition. $\Delta \varphi_{h,h+1\_k}$ indicates the change value of the phase offset between the *h*th intersection and the *h* − 1th intersection at the *k*th cycle and the *k* − 1th cycle, $\varphi_{h,h+1\_k}$ represents the phase offset value between the *h*th and *h* − 1th intersections at the *k*th cycle. The change in phase offset value between the *h*th and *h* − 1th intersections in the *k*th cycle is shown by the slope $\theta_{h,h+1\_k}$.

## 6. The Solution Algorithm

This section aims to derive the optimal solution for achieving smooth transitions in phase offset, as shown in (54)–(56). The nonlinear control system involves coordinating the optimization of multiple parameters. Traditional derivative-based methods for solving this operation are excessively complex and prone to finding only local optimal instead of the global optimal solution. Therefore, this paper will employ the Deep Q-Learning to solve for the optimum value solution.

Deep Q-learning is a reinforcement learning algorithm that utilizes a Deep Neural Network (DNN) with Q-learning. This section introduces reinforcement learning, Q-learning, and Deep Q-learning algorithms. Reinforcement learning algorithms are a type of machine learning algorithm in which an agent learns how to behave in an environment by taking actions and receiving rewards based on those actions. The objective of the agent is to maximize the expected future rewards. The state-space includes all possible environmental states, while the action-space comprises all actions that an agent can perform on the environment. At any given time *t*, the state of the environment, the action taken, and the reward received are denoted by *S_t_*, *A_t_*, and *R_t_*, respectively. The Q-Learning algorithm learns the maximum expected future reward for taking any action in a given environmental state, known as the Q-value. The Q-value for a state-action pair at time-step *t*, *Q*(*S_t_*,*A_t_*), is defined as the expected future reward of taking action *A_t_* in state *S_t_* at time-step *t*.

(57) $$Q\left(S_{t},A_{t}\right)=E\left[R_{t+1}+\xi R_{t+2}+\xi^{2}R_{t+3}+\xi^{3}R_{t+4}+\cdots \right]\left|\left(S_{t},A_{t}\right)\right.$$

where ξ is the discount factor, which should be between 0 and 1. This factor ensures greater significance is given to immediate rewards as opposed to those far off in the future.

The equation can be recursively written as follows:

(58) $$\begin{array}{ll} Q\left(S_{t},A_{t}\right) & =E\left[R_{t+1}+\xi \left[R_{t+2}+\xi R_{t+3}+\xi^{2}R_{t+4}+\cdots \right]\right]\left|\left(S_{t},A_{t}\right)\right.\\ & =E\left[R_{t+1}+\xi Q\left(S_{t+1},A_{t+1}\right)\right]\left|\left(S_{t},A_{t}\right)\right. \end{array}$$

The optimal *Q*-value *Q*(S^*^_t_,*A_t_*) is calculated as follows:

(59) $$Q\left({S^{*}}_{t},A_{t}\right)=E\left[R_{t+1}+\xi \max_{a\in A_{t+1}}Q\left(S_{t},a\right)\right]\left|\left(S_{t},A_{t}\right)\right.$$

The agent learns *Q*(S^*^_t_,*A_t_*) using the epsilon-greedy strategy. This means that the algorithm explores the state space by taking random actions with a probability of *є*, and selects the action with the highest Q-value among all possible actions in a given state with a probability of 1 − *є*. The value of *є* is initially set to 1 and gradually reduced as the training progresses. This ensures that the agent’s actions are more random at the beginning of the training when little is known about the environment, and less random towards the end when it predominantly chooses an action with the maximum *Q*-value corresponding to its state. In other words, it chooses to exploit the information gathered about the environment during training.

At each time step, the agent interacts with the environment to generate a sample (*S_t_*,*A_t_*,*R_t_*_+1_,*S_t_*_+1_,*A_t_*_+1_), which is then used to calculate the updated *Q*-value as follows:

(60) $$Q^{\prime }\left(S_{t},A_{t}\right)=R_{t+1}+\xi \max_{a\in A_{t+1}}Q\left(S_{t+1},a\right)$$

The expectation is calculated by taking an exponentially weighted average of many samples using a learning rate ς. This approach of calculating expectation by taking the average of a large number of samples is called sample-based learning and is based on the Law of Large Numbers.

(61) $$Q\left({S^{*}}_{t},A_{t}\right)=\left(1-\varsigma \right)Q\left(S_{t},A_{t}\right)+\varsigma Q^{\prime }\left(S_{t},A_{t}\right)$$

Equations above can be combined and re-arranged to give the following:

(62) $$Q\left({S^{*}}_{t},A_{t}\right)=Q\left(S_{t},A_{t}\right)+\varsigma \left[R_{t+1}+\xi \max_{a\in A_{t+1}}Q\left(S_{t+1},a\right)-Q\left(S_{t},A_{t}\right)\right]$$

**Figure 16:**
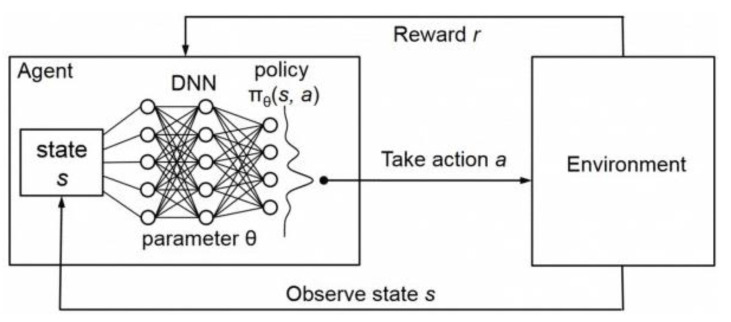
The DQN architecture.

Q-learning enables an agent to learn an optimal policy, denoted as π*, which allows the agent to choose an action in any given environment state, *S_t_*, that will optimize its future expected rewards. This is achieved by selecting the action with the maximum Q-value among all possible actions in the state. However, in most practical use cases, the state-action space of the environment is significantly large, making it nearly impossible to learn all required *Q*-values within reasonable time and resource constraints. To address this issue, a Deep Neural Network (DNN) is employed to estimate the *Q*-value function. The DNN extracts features from the states and generates *Q*-values. The DQN (Deep Q-Learning Network) architecture, is depicted in Figure 16.

The function *Q*(*S*,*A*) is approximated by $\hat{Q}\left(S,A,w\right)$, where *w* is the parameter vector for the DNN that minimizes the least mean square error between the two functions. The DNN’s loss function is defined as follows:

(63) $$J=\frac{1}{2}{\left[Q\left(S,A\right)-Q\left(S,A,w\right)\right]}^{2}$$

To minimize the aforementioned function, we calculate the gradient of the function *w*, *r*, *t*. parameter vector *w*:

(64) $$\nabla J=\left[Q\left(S,A\right)-Q\left(S,A,w\right)\right]\nabla Q\left(S,A,w\right)$$

The gradient points indicate the direction of *J*’s increase. Vector w is updated in the direction opposite to ∇J as per the equation below *w_t_*_+1_ = *w_t_* − ς∇J.

(65) $$w_{t+1}=w_{t}-\varsigma \left[Q\left(S_{t},A_{t}\right)-Q\left({\hat{S}}_{t},A_{t},w_{t}\right)\right]\nabla Q\left({\hat{S}}_{t},A_{t},w_{t}\right)$$

As the true *Q*-value *Q*(*S_t_*,*A_t_*) is unknown, we use a sample predicted by the current DNN.

(66) $$Q\left(S_{t},A_{t}\right)=R_{t+1}+\xi \max_{a\in A_{t+1}}Q\left(S_{t+1},a\right)$$

(67) $$w_{t+1}=w_{t}-\varsigma \left[R_{t+1}+\xi \max_{a\in A_{t+1}}Q\left(S_{t+1},a\right)-\hat{Q}\left(S_{t},A_{t},w_{t}\right)\right]\nabla \hat{Q}\left(S_{t},A_{t},w_{t}\right)$$

When performing a TD-update [48] in Deep Q-Learning, it is important to consider several issues. One of these issues is that the agent may forget previous experiences as training progresses. Another issue is that training data generated by consecutive interactions with the environment can be highly correlated, leading to a biased learned model. To address this, experience-replay is used as a methodology during Deep-Q learning. An experience is a tuple [*S_t_*, *A_t_*, *R_t_*_+1_, *S_t_*_+1_, done] that is generated through interaction with the environment. The ‘done’ value is set to true if the training episode is complete, otherwise it is set to false. Experiences are stored in a replay buffer and training is performed by replaying a batch of random experiences from the buffer. This ensures that the model learns from the same experience multiple times. Furthermore, the use of randomly selected training batches helps to eliminate any potential model bias that may result from training on consecutively generated samples.

### 6.1. Scenario and Simulation Environment

**Figure 17:**
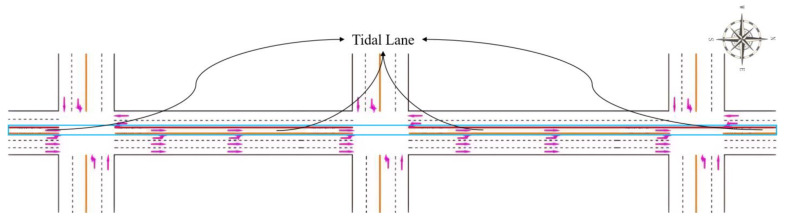
The traffic statistics for arterial intersections in the tidal lane, that is, the direction of the tidal lane is from south to north.

**Figure 18:**
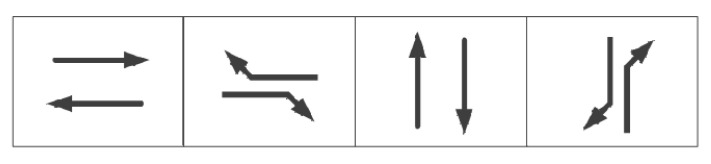
The phase diagram of arterial signal.

**Figure 19:**
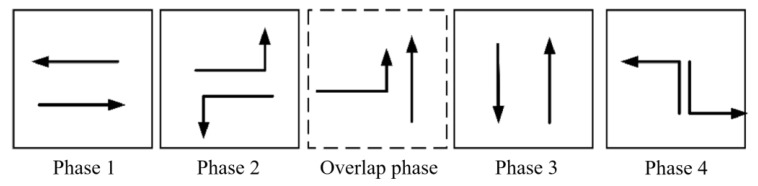
Overlap phase sequence scheme of arterial intersections.

**Figure 20:**
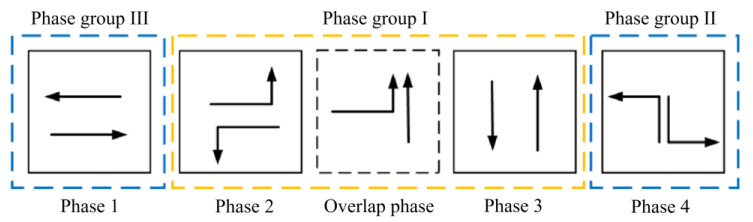
Overlap phase sequence scheme.

For the exemplification analysis, three successive intersections within a city have been selected. Figure 17 represents a plan view of arterial intersections. The tidal lanes shown in Figure 17 are intended for southbound traffic that is moving in a straight line, meaning that the direction of the tidal lanes is from south to north. The length of each road section in the tidal lane of arterial intersections is between 500 and 1000 m. Also, the traffic crossing considered in this paper is a four-way intersection with directions to the North, South, East and West. The intersection consists of one dedicated left-turn lane and one straight lane in each direction: East, West, and North. In the South direction, there is one tidal lane, one dedicated left-turn lane, and two straight lanes. The initial phase sequence scheme for the arterial intersection is shown in Figure 18, utilizing four-phase signal timing. To improve efficient arterial capacity and smooth traffic flow, an improved phase-phase sequence scheme is employed. This is accomplished through the adoption of an overlap phase scheme, as shown in Figure 19 and Figure 20, in accordance with the phase sequence scheme for the tidal lane scenario outlined in Section 3. The traffic signal at the intersection is controlled by an agent based on Deep-Q learning. The agent receives information about the state space, including the various opening states of the tidal lanes, and a reward signal phase offset from the simulation environment. Based on the current traffic state, the agent makes decisions.

### 6.2. State Space

The state-space for this paper was constructed by various opening states of the tidal lanes (towards the traffic-signal), controlled by a dedicated traffic light, to a lane-group. Each lane-group is further divided into three types. The first is the tidal lane (TL) type, consisting of the first 1 lane. The opening situation of the arterial intersection tidal lane is divided into two categories, i.e., synchronous opening and asynchronous opening (where α = l or 0, and the value 1 represents the opening of the tidal lane, while the value 0 indicates its closure). The second is the left-turn lane (LTL) type, consisting of the second 1 lane. The third is the straight lane (SL) type, consisting of the third 2 lane. Also, each lane-group is further divided into 10 lane-cells. Lane-cells near the traffic intersection are configured to be smaller in length as compared to those further away the intersection. This is because vehicles are slow moving and closely spaced near the intersection than away from it (where they can be fast moving and/or sparsely inter-spaced). Thus, four lanes belonging to an arm of a traffic intersection were divided into two lane-groups with 10 lane-cells, accounting for 20 lane-cells per traffic intersection arm and 20 × 4 = 80 lane-cells for the complete traffic intersection. State space was implemented as a vector of 80 integers wherein each element represented whether any vehicle was present or absent in a lane-cell.

(68) $$S=\left[e0,e1,e2,\cdots ,e79\right]$$

where $e\in \left\{0,1\right\}$.

### 6.3. Action Space

The action-space is defined as the set of all possible actions an agent can take. In this paper, each possible action is mapped to a traffic light phase, which corresponds to a possible state of the traffic signals controlling the intersection. The allowed traffic light phases, i.e., the action-space, are defined as follows: A = {NSA, NSLA, EWA, EWLA, OPNS}

NSA: North South Advance corresponds to the traffic-signal state allowing traffic to go from North direction to South and from South to North.

NSLA: North South Left Advance corresponds to the traffic-signal state allowing traffic to go from North towards the East and from the South towards West. Traffic in all other directions is stopped.

EWA: East West Advance corresponds to the traffic-signal state allowing traffic to go from East direction to West and from West to East. Traffic in all other directions is stopped.

EWLA: East West Left Advance corresponds to the traffic-signal state allowing traffic to go from East to South and from West towards North. Traffic in all other directions is stopped.

OPNS: Overlap Phase North South corresponds to the traffic-signal state allowing traffic to go straight from South to North at the phase sequencing scheme for tidal lane scenarios.

**Figure 21:**
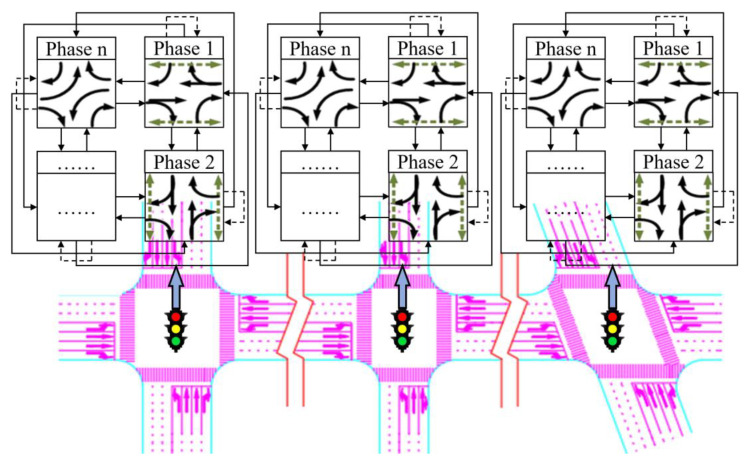
The diagram of action space.

To enable traffic to move in a particular direction, the corresponding traffic phase must be set to green. Traffic phases are set to green in 10-s intervals. To switch from one traffic phase to another, the previous traffic phase must be set to yellow for a duration of 4 s. The diagram of action space is shown in Figure 21. 

### 6.4. Deep Neural Network

**Figure 22:**
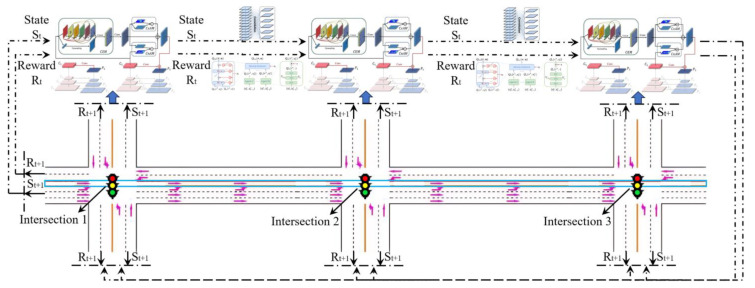
The diagram of deep neural network.

At the end of each control cycle, the neural network calculates the Q-values of all available actions for a given state space. The agent then selects the action with the highest Q-value, which is expected to produce the maximum reward. After executing the selected action, a new control cycle begins, and the agent continues to learn how to maximize the reward by interacting with the environment. To improve learning efficiency and reduce potential overestimation, our DQN algorithm utilizes a DNN with specific characteristics. The input layer takes a 1 × 80 binary vector. There are two hidden layers, each with 400 units and a RELU activation function. The output layer consists of 1 × 4 units with a LINEAR activation function, with each unit representing a traffic light phase. Figure 22 presents the diagram of deep neural network.

### 6.5. Reward Function

The reward of an action by the agent is calculated by comparing the average queue length and average delay of all vehicles in the simulation in the incoming lane, before and after the action is taken. If the action (traffic phase) resulted in vehicles to leave the intersection, it contributed towards decreasing the average queue length and average delay, resulting in a positive reward. However, an action that prohibits vehicles from crossing the intersection leads to an increase in the number of vehicles waiting at the intersection. This, in turn, results in a higher average queue length and average delay, resulting in a negative reward.

(69) $$R_{t}=0.9w_{t-1}-w_{t}$$

where *w_t_* is the distance (in meters) and time (in seconds) waited by all the vehicles in incoming lanes since the start of environment simulation until time step *t*. 0.9 is multiplied to *w_t_*_−1_ to stabilize learning. It results in an action to be considered favorable only if it significantly reduces the average queue length and average delay of vehicles compared to the previous time step.

### 6.6. Implementation Details

The implementation for training an adaptive solution algorithm for this paper includes three classes:

Model: The Model class implements the DNN to approximate the *Q*-value function. The model is implemented using the Keras v-2.2.4 with Tensorflow v-1.12.0 backend.

VissimEnv: This class encapsulates the simulation of a traffic intersection as described in previous sections. It uses the Traffic Control Interface (TCI) to retrieve statistics related to simulated objects and to manipulate their behavior during training. The class provides interfaces that the agent can use to control the simulation.

Step 1: Start environment simulation.

Step 2: Reset environment simulation

Step 3: Perform action on the environment and receive rewards for it

Step 4: Query existing environment state

Step 5: Query statistics, such as, cumulative delay of all vehicles in a simulation and cumulative intersection queue size at any given time in a training episode.

Agent: This class implements an adaptive agent that interacts with the VissimEnv class to train an instance of the Model class for approximating the *Q*-value function. The DQN algorithm is used for selecting actions to be taken on the environment. This paper describes an agent that implements a coordinated method for tidal lanes that is proposed to optimize the phase offset at arterial intersections for smooth and rapid transitions to enable a comparison of performance between synchronous and asynchronous traffic signal transition control strategies. The DQN algorithm implemented in the class has the following key implementation details.

Step 1: A cyclic replay buffer with a size of 50,000 is created to store the agent’s experiences with the environment. Each experience is represented by a tuple [*S_t_*, *A_t_*, *R_t_*_+1_, *S_t_*_+1_, done], where “done” is set to true if the training episode is complete, and false otherwise.

Step 2: At each time step, a batch of 100 random experience tuples is chosen and prepared for training. This approach of learning from cached experiences is called experience replay. The replay logic is implemented as follows:

Step 2.1: *Q*-values for all actions corresponding to state *S_t_* for all samples in the batch are obtained by predicting them from the DNN model.

Step 2.2: The *Q*-values corresponding to *A_t_* for each sample in the batch is updated as per following rules:

Step 2.2.1: If the episode has ended i.e., done = true, $Q^{\prime }\left(S_{t},A_{t}\right)=R_{t+1}$ ELSE.

Step 2.2.2: Predict all *Q*-values corresponding to state *S_t_*_+1_ and find the maximum amongst them.

Step 2.2.3: Update *Q*-value corresponding to *A_t_* action for state *S_t_* using Equation (59).

Step 3: The training input comprises an array of *S_t_* in the sampled batch, while the training target consists of the updated *Q*-values as demonstrated above.

Step 4: Using the same DNN to predict *Q*-values for preparing training data and modifying weights during training can cause significant oscillations in the training process. This situation is similar to chasing a moving target. To mitigate this issue, our DQN algorithm maintains an additional Target DNN, which is used to predict *Q*-values corresponding to *S_t_*_+1_. The weights of this network remain constant for 2000 episodes, after which they are copied from the trained DNN to the target DNN.

## 7. Results

To evaluate the effectiveness of the traffic signal transition control method, this section utilizes VISSIM 4.30 simulation software for verification. Firstly, it is necessary to conduct statistical analysis of the traffic volume and road channelization at the arterial intersections. Subsequently, the arterial intersection plan should be created using AUTOCAD 2023 software, and traffic flow statistics should be obtained through Python on VISSIM simulation software for secondary development. Following this, the arterial intersection signal transition scheme can be verified through using “VISSIM + Python” simulation. To construct a network, the following steps must be performed.

Step 1: Edge Mode: Creation of four, two-way edges of length 500~1000 m with a common junction.

Step 2: Inspect mode:

Step 2.1: Assign junction IDs as JW, JN, JE, JS and TL for traffic junctions corresponding to each direction and traffic light respectively.

Step 2.2: Assign edge IDs as N2TL, TL2N, S2TL, TL2S, E2TL, TL2E, W2TL and TL2W based on the direction of the edges.

Step 3: Traffic Light Mode:

Step 3.1: A traffic transition signal light is added to junction OPNS.

Step 3.2: Traffic phases NSA, NSLA, EWA and EWLA are configured with one green and one yellow phase for each, making a total of eight phases with IDs 0 through 7.

Step 4: Connection Mode: Connections are edited to ensure that the tidal lane of a four-lane edge could only switch turn left. The second lane is left lane that could only turn left. The rest of the two lanes could only go straight.

### 7.1. Collection of Data on Arterial Intersections

To test the efficacy of the model proposed in this paper, the traffic flow data at the intersections 1–3 are presented in Table 6, Table 7 and Table 8.

### 7.2. The Results of Signal Transition Control Based on OAS Deep Q-Learning

This section evaluates the performance of OAS Deep Q-Learning is compared with those of two categories of the baseline methods: traditional methods (including MaxPressure [49] and FixedTime [50]) and the Multi-Agent Deep Reinforcement Learning (MARL) [51], Meta Variationally Intrinsic Motivated Reinforcement Learning (MetaVIMRL) [52], and Cooperative Multi-Agent Deep Q-Network (CMDQN) [53] based on the training results. For fair comparisons, the parameters and simulation conditions of the traditional methods and the DQL methods [51,52,53] are the same. Table 9 presents the parameters of OAS Deep Q-Learning with reinforcement learning network as mentioned in this paper.

**Figure 23:**
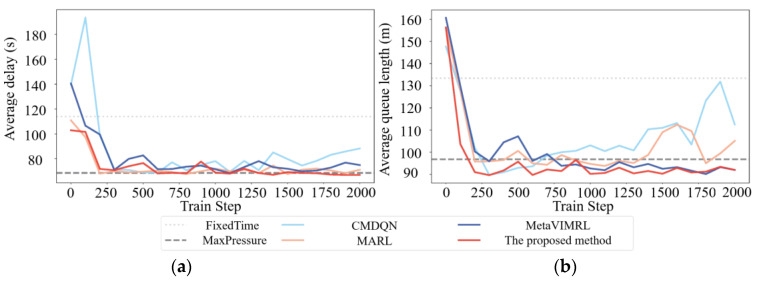
The comparison training results of 6 algorithms. (**a**) The comparison results of average delay with 6 algorithms; (**b**) The comparison results of average queue length with 6 algorithms.

As shown in Figure 23, the average queue lengths of vehicles on the traffic intersection experience an overall decrease with every train step as the training progresses. Another statistic, average delays, also shows progressive decrease during training. Note that the better performance of our traffic-signal controller agent on both counts (average queue lengths and average delays) as training progresses proves that our OAS Deep Q-Learning model is successfully learning to adapt the transitions of phase offsets in various tidal lane scenarios to the different opening states of the tidal lanes at related intersections. The proposed method outperforms other methods in all tidal lane signal transition scenarios. As expected, the proposed method has better performance than other Deep Q-learning methods. The performance evaluation metrics used are average delay and average queue length. The proposed method has the smallest average delay value of 73.12 and the shortest average queue length value of 92.35. The proposed method outperforms other Deep Q-learning methods in terms of both data efficiency and performance.

**Figure 24:**
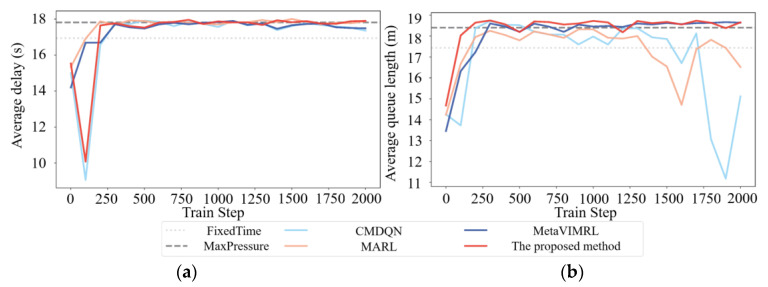
The Convergence of 6 algorithms. (**a**) The convergence of average delay with 6 algorithms; (**b**) The convergence of average queue length with 6 algorithms.

In general, the desired outcome of the training curves is convergence, as the agent learns from historical experience. Figure 24 demonstrates that the training curves of the proposed method and all DQL baseline algorithms [48,49,50] exhibit an upward trend before converging. Among them, the training curves of CMDQN and MARL in Figure 24b fluctuate more, but the performance of CMDQN is worse. This is because CMDQN and MARL algorithms are similar in that each agent updates its own policy independently, which makes the environment non-smooth. Furthermore, it is important to note that non-smoothness can result in slower and less stable training. As anticipated, MetaVIMRL outperforms MARL due to the fact that each local agent takes into account policy information from its surroundings, thereby mitigating the impact of partial observability on convergence. Conversely, OAS Deep Q-Learning exhibits the best performance as it demonstrates the fastest convergence rate, the smoothest convergence curve, and the highest reward. In other words, Figure 24 shows that the average queue lengths and average delays improve with the training process. However, the method proposed in this paper outperforms the training process by converging faster and exhibiting less fluctuation. 

**Figure 25:**
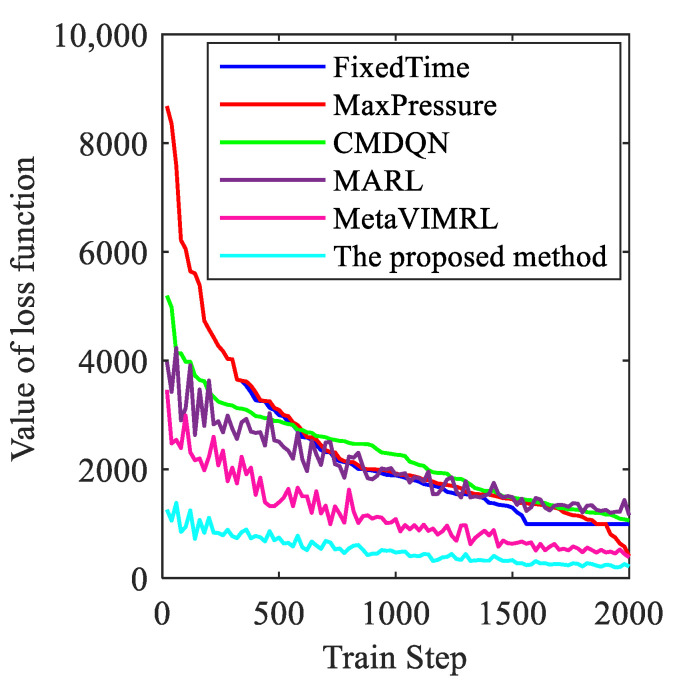
Variation of loss function for the 6 algorithms.

The loss function metric indicates the performance of the model’s training. A smaller value of the loss function indicates better training. Figure 25 shows the change in the loss function for each algorithm during the training process.

Figure 25 shows that all algorithms eventually converge. FixedTime and MaxPressure have a larger loss function. At a training step size of approximately 1520, FixedTime’s loss function converges, with an average of 2384.56. Meanwhile, MaxPressure’s loss function has an average of 2449.73. The proposed method has small fluctuations in the loss function, which is converged at a training step size of approximately 1000. The average value of loss function is 519.37. The performances of the MARL, MetaVIMRL, and CMDQN algorithms differ significantly from the algorithms proposed in this paper. This is mainly due to differences in their network training and the complexity of their training parameter settings. The loss function of MARL converges at around 1500 training steps, with an average value of 2034.40. The loss function of MetaVIMRL converges at around 1470 training steps, with an average value of 1179.90. The loss function of CMDQN is 2280.27. At a training step size of approximately 1470, the MetaVIMRL loss function converges, with an average value of 1179.90. The CMDQN loss function, on the other hand, has an average value of 2280.27. Thus, OAS Deep Q-Learning model is more adaptable than the other algorithms.

To evaluate the computational efficient of the proposed method, we compared it with five other methods using runtime as the evaluation metric. Table 10 shows that runtime is chosen as the evaluation metric to compare the computational cost of all methods. After several experiments, the proposed method can avoid the complex learning process of other deep Q learning methods and outperforms traditional methods and other deep Q learning in terms of runtime, with the shortest average runtime of 32 s. It can also perform high-level computation with real-time input of parameters.

To enhance the verification of the robustness and reliability of the operation results, a comparison between the algorithm presented in this paper and other algorithms is necessary. It is important to note that achieving optimal control in complex environments requires sufficient access to each state-action space, which may lead to overfitting and result in poor control performance. However, to ensure robust performance on untested states, the agent must either be trained on a large dataset that includes as many state-action variations as possible or the state and action space must be simplified. Both approaches, however, may impact the controller’s convergence and optimality. Therefore, it is necessary to reduce the dimensionality of the state space while increasing the action space to achieve improved control performance.

**Figure 26:**
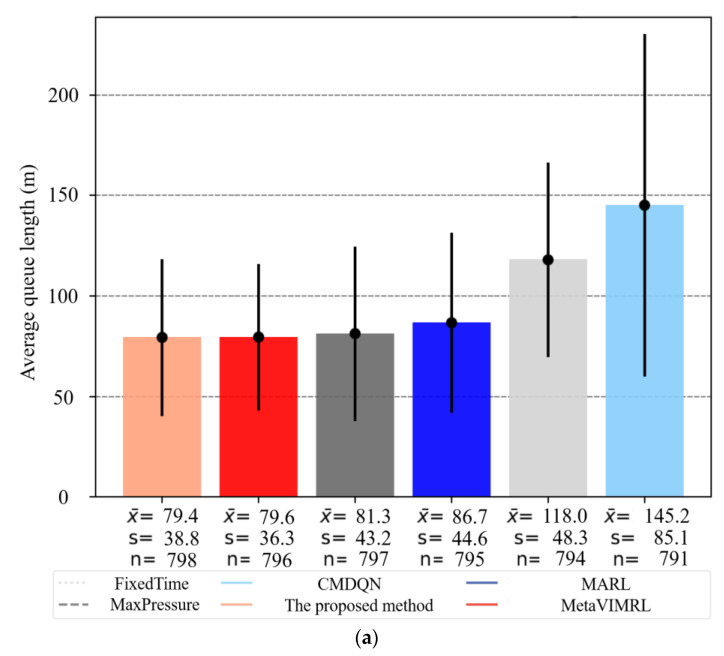

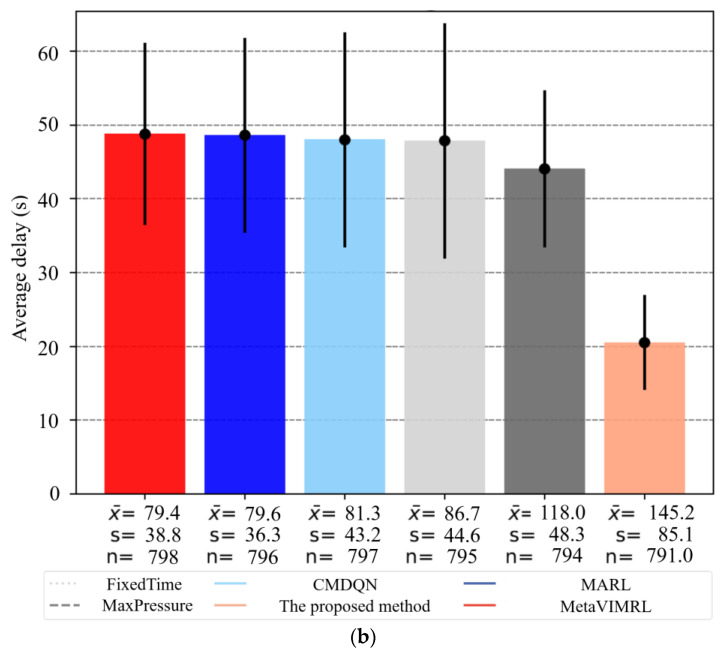
The box plot results of the proposed method and other algorithms. (**a**) The box plot’ average queue length results of 6 algorithms; (**b**) The box plot’ average delay results of 6 algorithms.

A box plot (shown in Figure 26) displays the data of the proposed method and other algorithms. In Figure 26, $\bar{x}$ is represented the average value. *s* is represented the standard deviation. *n* is represented the number of experimental runs for obtaining the best value. The comparison of the average queue length and average delay reveals a correlation between the chosen performance metrics; shorter queue length result in smaller delays. Figure 26a illustrates that the algorithm proposed in this paper has shorter queue lengths than the other algorithms by 7.14%, 14.29%, 21.43%, 39.13%, and 50.00%, respectively. Figure 26b illustrates that the algorithm presented in this paper has a smaller delay time compared to other algorithms by 59.18%, 58.33%, 57.45%, 56.52%, and 53.49%, respectively.

The sensitive results were verified to be statistically significant using left-tailed hypothesis testing. To perform hypothesis testing on paired means, the absolute value of measurements obtained for a particular simulation by executing the five algorithms are subtracted from absolute values of measurements obtained by executing OAS Deep Q-Learning. During execution, the DNN model is evaluated for optimal policy π* for all the 10,000 simulations and the performance statistics recorded. Policy π* evaluation involves taking actions that have maximum Q-value in the state where the agent is at any time-step *t*. Similarly, the performance statistics generated on the execution of five algorithms is also recorded.

(1) Average queue length

(70) $$x_{\_}diff_{wt}=x_{-}OASDQL_{wt}-x_{\_}Othermethods_{wt}$$

(71) $$x^{-}diff_{wt}=\frac{1}{n}\sum x_{-}OASDQL_{wt}$$

We stated the Null and the Alternative hypothesis as below:

H_o_: There is no difference between the true mean *υ*_*OASDQL_wt_* and *υ*_*Othermethods_wt_* and the difference observed in the sample means *x*^−^*OASDQL_wt_* and *x*^−^*Othermethods_wt_* is a matter of chance. i.e.,

(72) $$\upsilon_{-}diff_{wt}=\upsilon_{-}OASDQL_{wt}-\upsilon_{-}Othermethods_{wt}=0$$

H_A_: Average queue length for all vehicles in traffic simulations executed using OAS Deep Q-Learning is on an average less than the same traffic simulations executed using the five algorithms. i.e., *υ*_*diff_wt_* < 0.

**Figure 27:**
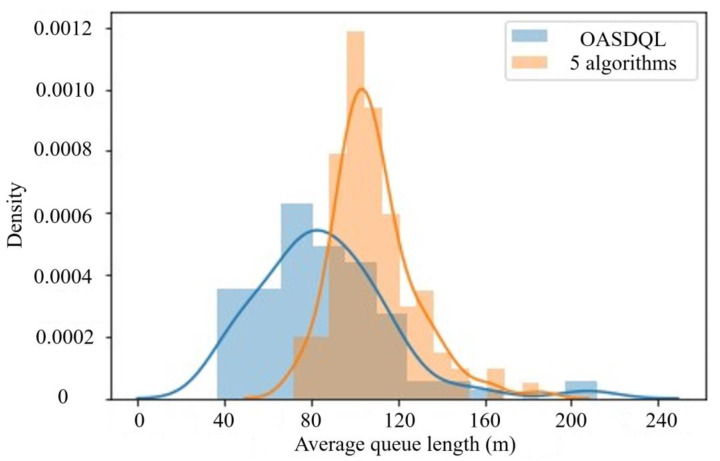
The sensitive results of average queue length.

Since the standard deviation of the actual distribution is not known the t-distribution was used for hypothesis testing. For a confidence level of 95% (significance = 0.05), degrees of freedom 99 (100 − 1) and left-tailed hypothesis testing. i.e., *t*_*c* = −1.66. Where *t*_*c* is the critical value of the t-score for a 10,000-sample mean, below which it is safe to reject the null hypothesis H_o_. Figure 27 presents the sensitive results of average queue length. Table 11 shows the sensitive parameters setting for average queue length.

*t*_*score* for the simulation sample captured above = −7.8. *p*-value < 0.00001. Since the calculated *p*-value << significance (0.05), we safely rejected H_o_.

(2) Average delay

(73) $$x_{\_}diff_{vqs}=x_{-}OASDQL_{vqs}-x_{\_}Othermethods_{vqs}$$

(74) $$x^{-}diff_{vqs}=\frac{1}{n}\sum x_{-}OASDQL_{vqs}$$

H_o_: There is no difference between the true mean *υ*_*OASDQL_vqs_* and *υ*_*Othermethods_vqs_* and the difference observed in the sample means *x*^−^*OASDQL_vqs_* and *x*^−^*Othermethods_vqs_* a matter of chance, i.e., as follows:

(75) $$\upsilon_{-}diff_{vqs}=\upsilon_{-}OASDQL_{vqs}-\upsilon_{-}Othermethods_{vqs}=0$$

H_A_: Average delay for all vehicles in traffic simulations executed using OAS Deep Q-Learning is on an average less than the same traffic simulation executed using the five algorithms. i.e., *υ*_*diff_vqs_* < 0.

**Figure 28:**
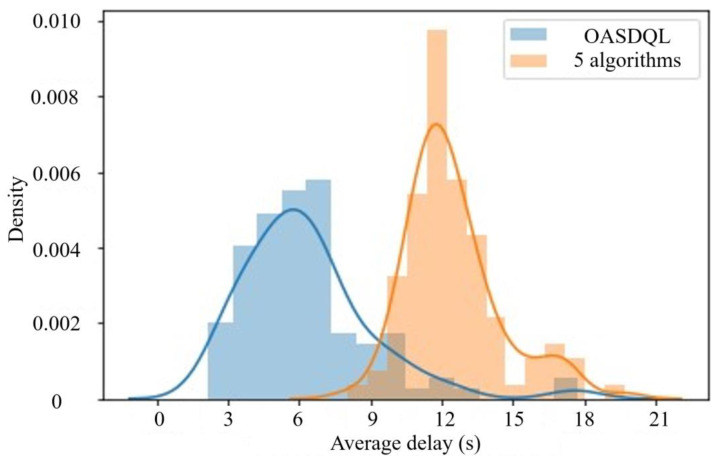
The sensitive results of average delay.

Since the standard deviation of the actual distribution is not known the t-distribution was used for hypothesis testing. For a confidence level of 95% (significance = 0.05), degrees of freedom 99 (100-1) and left-tailed hypothesis testing. i.e., *t*_*c* = −1.66. *t*_*c* is the critical value of the t-score for a 10,000-sample mean, below which it is safe to reject the null hypothesis H_o_. Figure 28 presents the sensitive results of average delay. Table 12 shows the sensitive parameters setting for average delay.

*t*_*score* for the simulation sample captured above = −29.8. *p*-value < 0.00001. Since the calculated *p*-value << significance (0.05), we safely rejected H_o_.

In summary, it has been demonstrated that the proposed method is not only viable but also more effective than the other five algorithms. The use of OAS Deep Q-Learning resulted in a great reduction in the average queue length of vehicles at the related intersections compared to the other five algorithms. Additionally, there is a reduction in the average delay at the related intersections.

In summary, Table 13 summarizes the results of the comparison between the proposed method and the previous algorithms shown in this work.

Table 13 shows that the proposed method is not only viable but also more effective than the previous algorithms simulated in this work. The proposed method has the smallest average delay value of 73.12 and the shortest average queue length value of 92.35. The proposed method outperforms other Deep Q-learning methods in terms of both data efficiency and performance. Compared with the previous algorithms, the proposed method has shortest queue length and the smallest delay time. The improvements in performance metrics with queue length and delay time are 26.39% and 56.99%, respectively. The proposed method has small fluctuations in the loss function, which is converged at a training step size of approximately 1000. The average value of loss function is 519.37. In other words, the method proposed in this paper outperforms the training process by converging faster and exhibiting less fluctuation. The proposed method has the shortest average runtime of 32 s. It can also perform high-level computation with real-time input of parameters.

### 7.3. Singal Transition Scheme

#### 7.3.1. Signal Transition Scheme Solution

The common cycle scheme for arterial intersections is determined based on the signal transition scheme for each of the three intersections, selecting the transition cycle scheme with the largest cycle. Table 14, Table 15, Table 16, Table 17 and Table 18 show the signal timing scheme for each intersection using the common cycle.

According to Table 14, Table 15, Table 16, Table 17 and Table 18, it is evident that diverse opening times for signal transition result in different signal transition schemes. When the signal transition commences after the tidal lane is opened or simultaneously with its opening, the signal cycle preceding the transition remains unchanged, and the original phase-phase sequence scheme for the intersection is maintained. When the signal transition begins following the opening of tidal lanes, a change in the number of lanes in the direction of tidal traffic occurs. This change results in an alteration of the signal cycle, as well as the phase-sequence scheme. Thus, the same phase-sequence scheme as the signal scheme following the transition is adopted. The tidal lanes are opened prior to the transition, therefore leading to this outcome.

#### 7.3.2. Traditional Signal Transition Scheme

The opening of the tidal lane may cause traffic flow conflicts. The traditional signal transition control scheme has some shortcomings in adjusting the intersection signal phase-phase sequence scheme. To better validate the effectiveness of the proposed model in this paper, the traditional signal transition scheme is optimized and the original intersection phase-sequence scheme is adjusted, which is shown in Table 19 and Table 20.

Compared to the signal transition scheme presented in this paper, the conventional scheme fails to modify the signal period subsequent to the signal transition process which eventually leads to elongation of the signal period at the intersection. However, this paper’s scheme adjusts the signal period, resulting in optimal signal operations.

### 7.4. Simulation Verification

**Figure 29:**
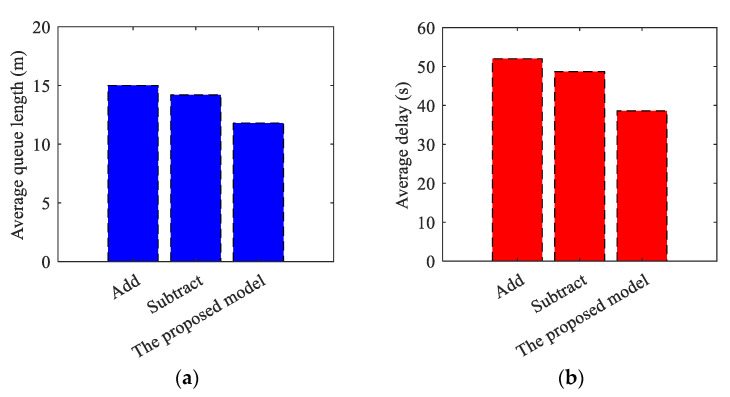
The comparison results for synchronous transition. (**a**) Average queue length for synchronous transition; (**b**) Average delay for synchronous transition.

**Figure 30:**
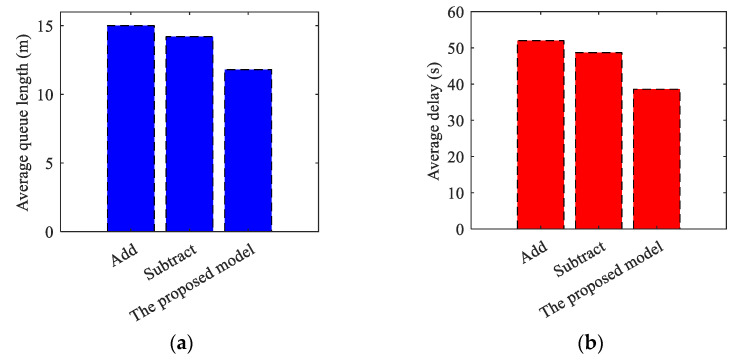
The comparison results for asynchronous transition. (**a**) Average queue length for asynchronous transition; (**b**) Average delay for asynchronous transition.

**Figure 31:**
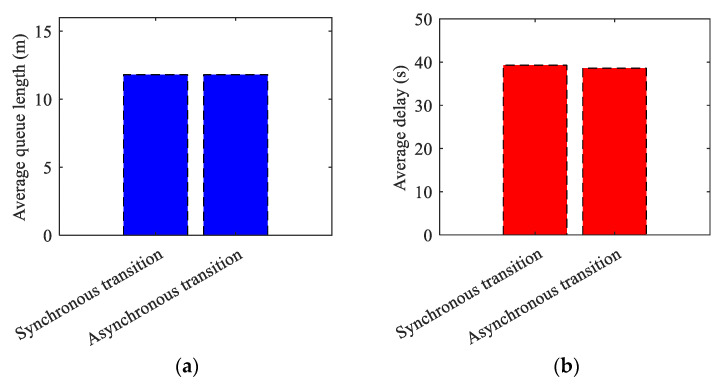
The comparison results for synchronous and asynchronous transitions. (**a**) Average queue length results comparison of synchronous and asynchronous transitions; (**b**) Average delay results comparison of synchronous and asynchronous transitions.

This paper compares and analyzes the signal transition control method with the traditional transition control method to determine the optimal opening scheme for tidal lanes at arterial intersections. The effectiveness of the proposed model is also verified in Table 21, Table 22 and Table 23 and Figure 29, Figure 30 and Figure 31.

(1) Synchronous Transition Verification

**Table 21:** Simulation Results of Synchronous Transition.

	Simulation Time (s)	Average Queue Length (m)	Average Delay (s)
Add	0–2000	15.5	54.0
Subtract	0–2000	14.7	51.3
The proposed model	0–2000	11.8	39.3

(2) Asynchronous Transition Verification

**Table 22:** Simulation Results of Asynchronous Transition.

	Simulation Time (s)	Average Queue Length (m)	Average Delay (s)
Add	0–2000	15.0	52.0
Subtract	0–2000	14.2	48.7
The proposed model	0–2000	11.8	38.6

(3) Simulation results comparison of synchronous and asynchronous transitions.

**Table 23:** Simulation Results of Synchronous and Asynchronous Transition.

	Simulation Time (s)	Average Queue Length (m)	Average Delay (s)
Synchronous transition	0–2000	11.8	39.3
Asynchronous transition	0–2000	11.8	38.6

The results of the simulation demonstrate that the algorithm introduced in this paper decreases the average queue length indicator of the related intersections by 23.87% and 19.73%, and the average vehicle delay indicator by 27.22% and 23.39%, respectively, when comparing synchronous transitions to the conventional Add and Subtract algorithms. The related intersections’ average queue length decreased by 21.33%. The average vehicle delay indicator also decreased by 25.77% and 20.74% respectively during asynchronous transition. Additionally, the average delay decreased by 1.78% in asynchronous compared to synchronous transition. The proposed algorithm outperforms the traditional algorithm in both synchronous and asynchronous transitions. Meanwhile, it is apparent from the conclusion that the asynchronous transition is more advantageous for maintaining traffic flow stability in the scenario of a tidal lane.

## 8. Conclusions

The proposed algorithm aims to optimize signal transitions for coordinated arterial intersection control under tidal lane scenarios. It calculates the signal transition scheme for each intersection on the arterial road using the single intersection signal transition method. The algorithm selects a common period for arterial intersections and analyses intersections that cannot be transitioned simultaneously. A model is constructed for smoothing phase offsets during signal transitions at arterial intersections. Firstly, the opening situation of the tidal lane at the arterial intersection is examined. Additionally, an investigation into various signal transition cycle scenarios is undertaken to determine the appropriate signal transition scheme. These schemes are categorized as synchronous or asynchronous transitions. This paper analyses phase offset transitions, considering various signal transition scenarios. It optimizes the phase offset calculation model to better suit the tidal lane situation and establishes a model for coordinating arterial control. It optimizes the phase offset calculation model to better suit the tidal lane situation and establishes a model for coordinating arterial control. The aim is to achieve smooth and rapid phase offset transitions. The optimal signal transition scheme is obtained by using OAS Deep Q-Learning to optimize and solve the issue. Simulation verification was conducted using VISSIM software and Python to evaluate the control effect of two distinct signal transition schemes. The results indicate that the asynchronous transition is more advantageous for maintaining traffic flow stability in the scenario of a tidal lane.

There are still many research contents left for future studies, and several of them are discussed in the following. A method is proposed for the tidal lane scenario, and the analysis of the scenario can be further refined. Additionally, it is necessary to investigate the specific phase offset between different transition cycles of adjacent intersection models.

## Figures and Tables

**Table 1:** Synchronous opening of tidal lanes.

	Intersection 1	Intersection 2	Intersection 3
Pre-transition cycle	1	1	1
Transition cycle I	1	1	1
Transition cycle II	1	1	1
Transition cycle III	1	1	1
Post-transition cycle	1	1	1

**Table 2:** Asynchronous opening of tidal lanes.

	Intersection 1	Intersection 2	Intersection 3
Pre-transition cycle	1	1	1
Transition cycle I	1	1	1
Transition cycle II	1	1	1
Transition cycle III	1	1	1
Transition cycle IV	1	1	1
Transition cycle V	1	1	1
Post-transition cycle	1	1	1

**Table 3:** Calculation Results of Signal Transition Cycle.

	Intersection 1	Intersection 2	…	Intersection 3
Pre-transition cycle	*C_A_*	*C_A_*	*C_A_*	*C_A_*
Transition cycle I	*C*_11_	*C*_21_	…	*C_n_*_1_
Transition cycle II	*C*_12_	*C*_22_	…	*C_n_*_2_
Transition cycle III	*C*_13_	*C*_23_	…	*C_n_*_3_
…	…	…	…	…
Transition cycle N	*C*_1*k*_	*C*_2*k*_	…	*C_nk_*
Post-transition cycle	*C_B_*	*C_B_*	*C_B_*	*C_B_*

**Table 4:** Synchronous Transition for Signal Scheme.

	Intersection 1	Intersection 2	Intersection 3
Pre-transition cycle	*C_A_*	*C_A_*	*C_A_*
Transition cycle I	*C*_1_	*C*_1_	*C*_1_
Transition cycle II	*C*_2_	*C*_2_	*C*_2_
Transition cycle III	*C*_3_	*C*_3_	*C*_3_
Post-transition cycle	*C_B_*	*C_B_*	*C_B_*

**Table 5:** Asynchronous Transition for Signal Scheme.

	Intersection 1	Intersection 2	Intersection 3
Pre-transition cycle	*C_A_*	*C_A_*	*C_A_*
Transition cycle I	*C*_1_	*C_A_*	*C_A_*
Transition cycle II	*C*_2_	*C*_1_	*C_A_*
Transition cycle III	*C*_3_	*C*_2_	*C*_1_
Transition cycle IV	*C_B_*	*C*_3_	*C*_2_
Transition cycle V	*C_B_*	*C_B_*	*C*_3_
Post-transition cycle	*C_B_*	*C_B_*	*C_B_*

**Table 6:** Traffic Flow at the Intersection 1.

Entrance Line	Flow Direction	Number of Lanes	α	Off-Peak Traffic Volume	Peak Traffic Volume
East	Straight	1	0	224	254
	Left	1	0	187	201
West	Straight	1	0	208	239
	Left	1	0	203	218
South	Straight	2	1	487	925
	Left	1	0	267	288
North	Straight	2	−1	256	312
	Left	1	0	256	277

**Table 7:** Traffic Flow at the Intersection 2.

Entrance Line	Flow Direction	Number of Lanes	α	Off-Peak Traffic Volume	Peak Traffic Volume
East	Straight	1	0	205	214
	Left	1	0	201	213
West	Straight	1	0	213	229
	Left	1	0	208	226
South	Straight	2	1	546	986
	Left	1	0	227	286
North	Straight	2	−1	267	323
	Left	1	0	176	183

**Table 8:** Traffic Flow at the Intersection 3.

Entrance Line	Flow Direction	Number of Lanes	α	Off-Peak Traffic Volume	Peak Traffic Volume
East	Straight	1	0	243	273
	Left	1	0	188	197
West	Straight	1	0	209	229
	Left	1	0	198	211
South	Straight	2	1	642	1046
	Left	1	0	223	310
North	Straight	2	−1	278	331
	Left	1	0	264	289

**Table 9:** The parameters of OAS Deep Q-Learning with reinforcement learning network.

Parameter	Value
Simulated time steps for each episode	2000
Replay memory size	50,000
Minibatch size	100
Target network update rate	0.001
Discount factor	0.9
Learning rate	0.0001

**Table 10:** Run times’ comparison with different methods.

Method	Run Times (s) for One Time	Run Times (s) for Two Time	Run Times (s) for Three Time	Run Times (s) for Four Time	Run Times (s) for Five Time
MaxPressure	672	684	668	690	675
FixedTime	411	410	421	418	416
CMDQN	181	192	184	186	184
MARL	165	165	164	164	163
MetaVIMRL	152	152	151	150	153
The proposed method	32	33	32	32	31

**Table 11:** The sensitive parameters setting for average queue length.

	OASDQL-5 Algorithms
*x* ^−^ *diff_wt_*	−572.404
*υ*_*diff_wt_*	733

**Table 12:** The sensitive parameters setting for average delay.

	OASDQL-5 Algorithms
*x* ^−^ *diff_vqs_*	−206.38
*υ*_*diff_vqs_*	69.03

**Table 13:** The summarization of the simulation results.

Method	Comparison Training Results of Average Delay	Comparison Training Results of Average Queue Length	Improvements in Performance Metrics with Queue Length	Improvements in Performance Metrics with Delay Time	The Average Value of Loss Function	Comparison Average Run Times (s)
MaxPressure	74.05	98.16	7.14%	59.18%	2449.73	677.80
FixedTime	74.05	98.16	14.29%	58.33%	2384.56	415.20
CMDQN	76.33	105.68	21.43%	57.45%	2280.27	185.40
MARL	75.40	96.21	39.13%	56.52%	2034.40	164.20
MetaVIMRL	75.22	95.49	50.00%	53.49%	1179.90	151.60
The proposed method	73.12	92.35	-	-	519.37	32.00

**Table 14:** Signal Transition Scheme at the Intersection 1.

Transition Scheme	Signal Cycle	*Y*	Phase I	Phase II	Overlap Phase	Phase III	Phase IV
*C_A_*	92	0.57	18	15	0	19	20
*C*_1_	95	0.60	18	16	0	20	21
*C*_2_	100	0.61	20	16	4	18	22
*C*_3_	106	0.64	20	16	2	25	23
*C_B_*	113	0.64	22	17	2	27	25

**Table 15:** Signal Transition Scheme at the Intersection 2.

Transition Scheme	Signal Cycle	*Y*	Phase I	Phase II	Overlap Phase	Phase III	Phase IV
*C_A_*	92	0.57	16	17	0	21	18
*C*_1_	95	0.58	17	17	0	22	19
*C*_2_	100	0.60	18	17	3	20	22
*C*_3_	106	0.64	18	17	2	26	23
*C_B_*	113	0.64	19	19	2	28	25

**Table 16:** Signal Transition Scheme at the Intersection 3.

Transition Scheme	Signal Cycle	*Y*	Phase I	Phase II	Overlap Phase	Phase III	Phase IV
*C_A_*	92	0.62	17	14	0	22	19
*C*_1_	95	0.52	18	14	0	23	20
*C*_2_	100	0.65	20	15	6	18	21
*C*_3_	106	0.67	21	15	2	25	23
*C_B_*	113	0.69	23	15	2	27	26

**Table 17:** Phase Offset for Synchronous Transition.

Transition Scheme	Phase Offset at the Intersection 1	Phase Offset at the Intersection 2	Phase Offset at the Intersection 3
*C_A_*	26	43	0
*C*_1_	28	45	0
*C*_2_	30	47	0
*C*_3_	31	50	0
*C_B_*	34	52	0

**Table 18:** Phase Offset for Asynchronous Transition.

Transition Scheme	Phase Offset at the Intersection 1	Phase Offset at the Intersection 2	Phase Offset at the Intersection 3
*C_A_*	26	43	0
*C*_1_	28	45	0
*C*_2_	30	48	0
*C*_3_	33	50	0
*C*_4_	36	54	0
*C5*	38	57	0
*C_B_*	39	60	0

**Table 19:** Add’s Signal Transition Scheme.

Transition Scheme	Signal Cycle	*Y*	Phase I	Phase II	Phase III	Phase IV
*C_A_*	92	0.62	17	14	22	19
*C*_1_	141	0.70	29	22	36	34
*C*_2_	141	0.70	29	22	36	34
*C*_3_	141	0.70	29	22	36	34
*C_B_*	117	0.70	24	18	29	26

**Table 20:** Subtract’s Signal Transition Scheme.

Transition Scheme	Signal Cycle	*Y*	Phase I	Phase II	Phase III	Phase IV
*C_A_*	92	0.62	17	14	22	19
*C*_1_	94	0.70	18	14	22	20
*C*_2_	94	0.70	18	14	22	20
*C*_3_	94	0.70	18	14	22	20
*C_B_*	117	0.70	24	18	29	26

## Data Availability

The data used to support the findings of this study are available from the first author upon request due to privacy reasons.
